# Acute cigarette smoke exposure leads to higher viral infection in human bronchial epithelial cultures by altering interferon, glycolysis and GDF15-related pathways

**DOI:** 10.1186/s12931-023-02511-5

**Published:** 2023-08-23

**Authors:** Ying Wang, Dennis K. Ninaber, Alen Faiz, Abraham C. van der Linden, Annemarie van Schadewijk, René Lutter, Pieter S. Hiemstra, Anne M. van der Does, Abilash Ravi

**Affiliations:** 1 PulmoScience Lab, Department of Pulmonology, Leiden University Medical Center, 2333 ZA Leiden, The Netherlands; 2 Respiratory Bioinformatics and Molecular Biology (RBMB), School of Life Sciences, University of Technology Sydney, Ultimo, Sydney, NSW 2007 Australia; 3 Department of Pulmonary Medicine, Amsterdam University Medical Center, University of Amsterdam, 1081HV Amsterdam, The Netherlands

**Keywords:** Bronchial epithelium, Cigarette smoke, Rhinovirus infection, Chronic obstructive pulmonary disease (COPD), RNA-Seq

## Abstract

**Background:**

Acute exacerbations of chronic inflammatory lung diseases, such as chronic obstructive pulmonary disease (COPD), are frequently associated with rhinovirus (RV) infections. Despite these associations, the pathogenesis of virus-induced exacerbations is incompletely understood. We aimed to investigate effects of cigarette smoke (CS), a primary risk factor for COPD, on RV infection in airway epithelium and identify novel mechanisms related to these effects.

**Methods:**

Primary bronchial epithelial cells (PBEC) from COPD patients and controls were differentiated by culture at the air–liquid interface (ALI) and exposed to CS and RV-A16. Bulk RNA sequencing was performed using samples collected at 6 and 24 h post infection (hpi), and viral load, mediator and l-lactate levels were measured at 6, 24 and 48hpi. To further delineate the effect of CS on RV-A16 infection, we performed growth differentiation factor 15 (GDF15) knockdown, l-lactate and interferon pre-treatment in ALI-PBEC. We performed deconvolution analysis to predict changes in the cell composition of ALI-PBEC after the various exposures. Finally, we compared transcriptional responses of ALI-PBEC to those in nasal epithelium after human RV-A16 challenge.

**Results:**

CS exposure impaired antiviral responses at 6hpi and increased viral replication at 24 and 48hpi in ALI-PBEC. At 24hpi, CS exposure enhanced expression of RV-A16-induced epithelial interferons, inflammation-related genes and CXCL8. CS exposure increased expression of oxidative stress-related genes, of GDF15, and decreased mitochondrial membrane potential. *GDF15* knockdown experiments suggested involvement of this pathway in the CS-induced increase in viral replication. Expression of glycolysis-related genes and l-lactate production were increased by CS exposure, and was demonstrated to contribute to higher viral replication. No major differences were demonstrated between COPD and non-COPD-derived cultures. However, cellular deconvolution analysis predicted higher secretory cells in COPD-derived cultures at baseline.

**Conclusion:**

Altogether, our findings demonstrate that CS exposure leads to higher viral infection in human bronchial epithelium by altering not only interferon responses, but likely also through a switch to glycolysis, and via GDF15-related pathways.

**Supplementary Information:**

The online version contains supplementary material available at 10.1186/s12931-023-02511-5.

## Introduction

Viral and bacterial respiratory infections are associated with the majority of exacerbations in chronic inflammatory lung diseases, such as chronic obstructive pulmonary disease (COPD) and asthma. Human rhinovirus (RV) is one of the most common viruses detected during exacerbations in patients with COPD and asthma [1]. Despite these associations, the pathogenesis of virus-induced exacerbations is incompletely understood, limiting the options for developing therapeutic strategies aiming at reduction of virus-induced exacerbations and thereby disease progression. The airway epithelium that lines human airways is the main target for initial RV infection. Airway epithelial cells are essential for host defense of the lungs. They provide a physical barrier, mount innate immune responses, mediate mucociliary clearance and orchestrate adaptive immune responses [2].

Airway epithelial cell cultures have been widely used to study RV infections as well as cigarette smoke (CS) exposures, the latter being the primary risk factor for development and progression of COPD. Studies have employed combinations of CS extract with RV exposure in submerged airway epithelial cells and showed impairment of anti-viral defenses with a subsequent increase in viral infection [3]. Another study used well-differentiated primary bronchial epithelial cells (PBEC) to study the effect of whole CS on RV infections and showed decreased gene expression of interferons and interferon response genes, and inhibitory effects of α-1 antitrypsin treatment [4]. However, it is currently unknown how the kinetics of early and late antiviral defenses are impaired by CS. In addition, other cellular host mechanisms induced by CS exposure that contribute to the increased viral load of CS-exposed airway epithelium are currently unexplored. In addition, studies have indicated that COPD patients may be more susceptible to RV infection [5], but it is not currently known how this is mediated.

To address the above, we used air–liquid interface (ALI)-cultures of well-differentiated PBEC from COPD and non-COPD donors that adequately represent the human airway epithelium in situ [6], combined with exposure to whole CS (gas and particulate phase) [7]. Using an unbiased RNA sequencing (RNA-Seq) approach, we aimed to explore signaling pathways regulated by CS exposure or RV infection, or their combination, and delineated possible mechanisms involved in the effect of CS exposure on RV replication. Furthermore, we performed cellular deconvolution analysis to identify possible changes in cellular composition in ALI-PBEC upon the different exposures. Importantly, we validated these findings in RV-A16-infected ALI-PBEC by comparing these to those obtained by analysis of a human RV-A16 challenge model. These findings provide further insight into the pathogenesis of virus-induced exacerbations and may help to explain higher RV infection levels of CS-exposed airway epithelium.

## Materials and methods

### Subjects

PBEC were isolated from tumor free, macroscopically normal resected lung tissue (bronchial rings) from patients undergoing surgery for lung cancer at the Leiden University Medical Center from COPD (GOLD stage II) donors and non-COPD controls, as described previously [8]. For CS and RV16 exposure experiments, 8 COPD and 8 non-COPD donors were included and viral load measurements and qPCR analysis were performed. From these donors, 7 COPD and 6 non-COPD donors were included for RNA-sequencing analysis; cells from one COPD and two non-COPD donors were excluded in the RNA-Seq analysis based on lower viral RNA levels in air-exposed ALI-PBEC. The experiments on interferon (n = 4), FCCP (n = 5), l-lactate (n = 3) treatments and GDF15 knockdown (n = 4) included only non-COPD donors. The baseline characteristics of COPD and non-COPD donors included for all experiments in this study, are provided in Table 1.

**Table 1:** Baseline characteristics of COPD and non-COPD donors

	WCS + RV-A16 exposure		RNA-seq analysis		IFNs treatment	FCCP treatment	L﻿-lactate treatment	GDF15 KD
	COPD	Non-COPD	COPD	Non-COPD	Non-COPD	Non-COPD	Non-COPD	Non-COPD
Subjects (n)	8	8	7	6	4	5	3	4
Sex ratio (Female/Male)	3/5	4/4	2/5	2/4	3/1	1/4	1/2	2/2
Smoke status	Ex-smoker (8)	Ex-smoker (8)	Ex-smoker (7)	Ex-smoker (6)	Ex-smoker (1) non-smoker (3)	Ex-smoker (2) non-smoker (2) Unknown (1)	Smoker (1) ex-smoker (1) non-smoker (1)	Smoker (1) ex-smoker (3)
Age (years)^#^	63.0 ± 2.6	65.4 ± 3.0	63.7 ± 2.8	64.3 ± 3.6	53.8 ± 3.1	58.2 ± 1.9	64.0 ± 1.2	68.3 ± 2.8
FEV1 (%predicted)^#^	65.6 ± 4.0	101.9 ± 6.7*	64.4 ± 4.4	99.7 ± 6.2*	103.0 ± 3.6	96.0 ± 3.5	101.3 ± 16.7	113.1 ± 6.9
FEV1 (L^)#^	1.9 ± 0.1	2.7 ± 0.2*	1.9 ± 0.1	2.9 ± 0.3*	3.3 ± 0.2	3.6 ± 0.3	3.0 ± 0.5	2.9 ± 0.2

Data showed with # are presented as mean ± SEM. COPD: chronic obstructive pulmonary disease; FEV_1_: forced expiratory volume in the first second. Statistical analysis was performed between COPD and non-COPD donors using Mann–Whitney non-parametric, unpaired t-test. *p value of < 0.05 was considered significant

### Exposure of primary bronchial epithelial cells

PBEC at passage 2 were seeded at a density of 40,000 cells/insert on 0.4 μm pore size 12-well Transwells (Corning Costar, Cambridge, USA). When confluent, cells were apically exposed to air for 4 weeks to initiate cell differentiation. ALI-PBEC from non-COPD donors (n = 8) and COPD donors (n = 8) were either exposed to whole CS from one cigarette or to room air as control for 4- 5 min, as previously reported [9]. Following exposure, smoke was removed by ventilation with air for 10 min. Based on the weight of the outlet filter before and after CS exposure, the box in which the 12-well plate was exposed received ~ 2.8 mg of CS particles. Following CS exposure, ALI-cultures were immediately infected by apical exposure with 200 µl of RV-A16 prepared in PBS (MOI 1) for 1 h. Next, PBEC were washed by PBS from the apical side to remove unbound virus and incubated for 6, 24 and 48hpi respectively. As controls, air- and CS-exposed ALI-PBEC were mock infected with PBS.

For interferon treatment, ALI-PBEC from non-COPD donors (n = 4) were stimulated with 10 ng/ml recombinant human interferon β (IFN-β, R&D Systems, Minneapolis, USA) or 10 ng/ml IFN-λ1 (PeproTech, London, UK). These interferons were added in the basal medium directly after CS exposure, followed by RV-A16 infection (MOI 1) for 1 h and the cells were harvested at 24hpi.

For FCCP treatment, ALI-PBEC from non-COPD donors (n = 3) were treated with 10 μM FCCP for 6 h from apical side of the inserts and lysed for qPCR analysis. FCCP (carbonyl cyanide 4-(trifluoromethoxy)phenylhydrazone), served as a positive control based on its ability to cause a decrease in mitochondrial membrane potential (MMP).

For l-lactate treatment, ALI-PBEC from non-COPD donors (n = 3) were treated with 10 mM sodium l-lactate (Sigma-Aldrich) in the basal medium for 10 min. The concentration (10 mM) was chosen based on the levels of l-lactate measured in CS-exposed ALI-PBEC at 24hpi. Next, cells were apically infected with RV-A16 (MOI 1) at room temperature, followed by an apical wash after 1 h and incubation for 24hpi.

The knockdown of *GDF15* was performed in the PBEC from non-COPD donors (n = 4) and after knockdown of *GDF15*, PBEC were cultured at ALI for 2 weeks and exposed to CS and RV-A16 with controls as described earlier and analyzed at 24hpi. *GDF15* knockdown in PBEC was performed based on Alt-R CRISPR-Cas9 System [Integrated DNA Technologies (IDT), Coralville, IA, USA] using a combination of electroporation and lipofectamine delivery methods.

Detailed procedures of cell culture, cigarette smoke exposure, RV-A16 stock preparations, TCID50 measurements, *GDF15* knockdown, RNA isolation, qPCR, ELISA, l-lactate measurements and lactate dehydrogenase (LDH) assay are provided in the Additional file 1: supplementary methods.

### RNA sequencing and analysis

A dataset of 104 samples was generated by mRNA sequencing (RNA-Seq, polyA enriched) using the Illumina NovaSeq600 sequencer, with 15 million paired-end reads per sample which was performed at GenomeScan (Leiden, the Netherlands). All samples had a RIN (RNA integrity number) score of > 6 as assessed by bioanalyzer (GenomeScan) and passed the FASTQ and alignment quality analysis with OmiWCSoft (Qiagen, Venlo, The Netherlands). Detailed description of RNA isolation, sequencing and analysis along with Gene Set Enrichment Analysis (GSEA) [14] and Ingenuity Pathway Analysis (IPA) are provided in Additional file 1: supplementary methods.

### Cellular deconvolution

The estimation in the ratio of epithelial cellular composition, including ciliated, secretory, basal and rare cells, was obtained by cellular deconvolution analysis of bulk RNA-Seq data as previously reported [10]. Briefly, based on minimized correlation and maximized distance between clusters in which genes with the most stable results across cohorts were selected and used to infer major cell type proportions, 400 genes from highly variable ones were selected and filtered according to the human Lung Cell Atlas v1.0 dataset [11] using AutoGeneS software. The RNA-Seq data was subsequently normalized to counts per million (CPM), and highly variable (HV) genes (N = 5000) were selected. Cellular deconvolution was performed based on the RNA-Seq dataset using the CIBERSORT support vector regression (SVR) method [12]. The relative proportion of cell types was compared between ALI-PBEC exposed to Air, CS, RV-A16 and CS combined with RV-A16 in COPD and non-COPD donors using 2-way ANOVA with Tukey’s or Bonferroni test.

### Measurement of mitochondrial membrane potential

The mitochondrial membrane potential (MMP) was measured using the live detection probe JC-10 (Enzo Life Sciences, Farmingdale, NY, USA). After WCS exposure or FCCP (10 μM) treatment for 10–30 min, 2.5 µg/ml JC-10 was added to the apical side of the inserts for 30 min in the cell incubator and PBS was used to remove JC-10. The inserts were then cut out and placed on a Superfrost Plus object glass (ThermoFisher) covered with a coverslip. Live cells were imaged with a Leica TCS SP8 confocal microscope (Leica Microsystems) at 630× original magnification. For mitoTEMPO (Sigma-Aldrich) treatment, a concentration of 50 μM, was added to the basal medium 4 h prior to CS exposure or FCCP treatment. The ratio of positive-stained cells from three random areas of each insert membrane of each independent experiment were quantified by ImageJ.

### In vivo RV-A16 challenge

The gene sets that were differentially expressed upon in vitro RV-A16 infection and/or CS exposure were analyzed using a previously published dataset [13] from nasal epithelial cells derived of non-smokers, non-COPD donors (n = 16; only placebo treated group included) after in vivo RV-A16 challenge. We compared interferon response, complement, necroptosis and inflammatory gene sets from our study with those expressed in nasal epithelial cells collected on days 3, 6, 9 and 13 post RV-A16 challenge and at baseline (one day before RV-A16 challenge). RNA was isolated from nasal brushes during a previously published study [13] containing predominantly nasal epithelial cells (~ 95%) and RNA sequencing (RNA-Seq) was performed in a similar platform as for the cultured ALI-PBEC. RNA-Seq of nasal brushes and its analysis was already published, which included the expression of interferon response gene set at day 3, 6, 9 and 14 compared to baseline (before RV-A16). The detailed information of in vivo RV-A16 challenge, method details and the inclusion and exclusion criteria of these donors can be found in a previous publication [13]. At each time point, two nasal brushings obtained were pooled and centrifuged at 1000×*g* (standard tabletop centrifuge) for 5 min at 4 °C. The pellet was dissolved in 1 ml of TRIzol (ThermoFisher) and stored at − 80 °C. After all samples were collected, they were thawed to room temperature, followed by addition of 200 µl of chloroform and inverted 10 times. The samples were centrifuged at 2000×*g* for 10 min at 4 °C. The aqueous phase was used to isolate RNA using protocol 5.3 from RNA XS extraction kit (Macherey–Nagel, Düren, Germany). The quality and concentration of the samples were assessed by using a fragment analyzer (Advanced Analytical Technologies, Inc, Ankeny, Iowa).

### Statistical analysis

Statistical analysis was performed in GraphPad PRISM 9.0 (GraphPad Software Inc., La Jolla, CA). Differences were explored by one-way or two-way ANOVA with Tukey’s test, paired or unpaired two-tailed, t-test. Data are shown as mean values ± SEM and differences at p values of < 0.05 were considered significant.

## Results

### Cigarette smoke-induced increase in viral infection can be prevented by interferon treatment

**Fig. 1:**
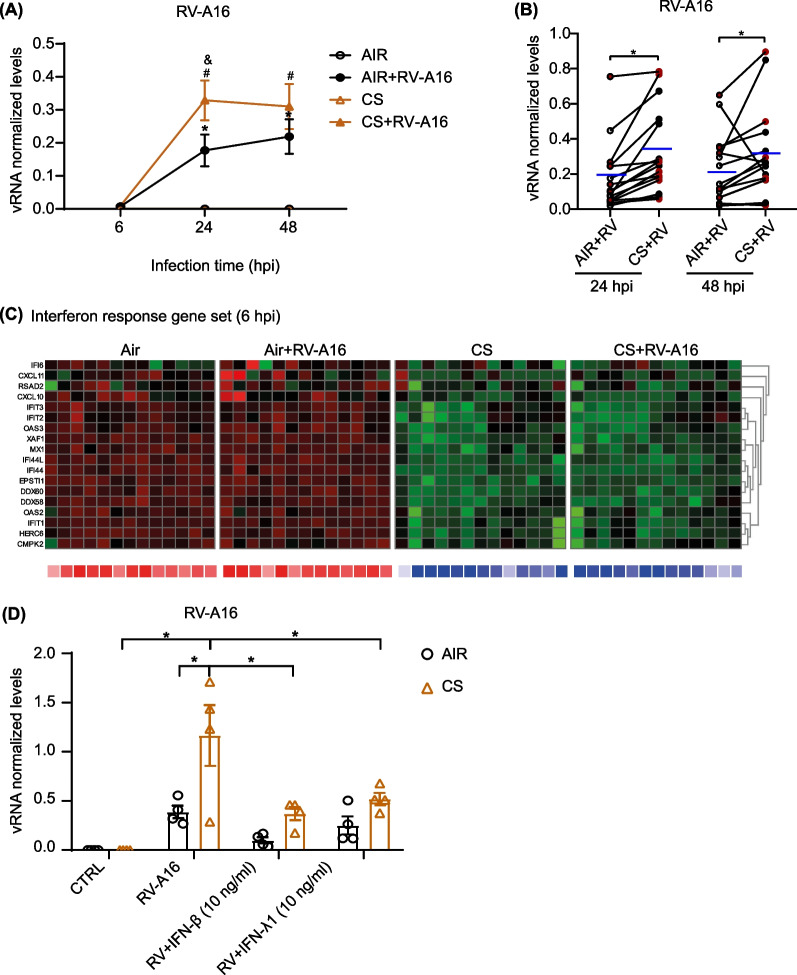
Effects of cigarette smoke exposure on rhinovirus infection and antiviral defenses in differentiated primary human bronchial epithelial cells. ALI-PBEC were exposed to CS or air control and then directly infected with RV-A16 (MOI 1) for 1 h and incubated for 6, 24 and 48 h. **A**, **B** Levels of RV-A16 vRNA at 6, 24 and 48hpi were measured by qPCR. Data are shown as target gene expression normalized for Ribosomal Protein L13a (*RPL13A*) and ATP synthase, h + transporting, mitochondrial F1 complex, beta polypeptide (*ATP5B*). Black symbols are data from non-COPD donors, red symbols from COPD donors. Data are mean values (blue line) ± SEM. n = 16 different donors. Analysis of differences was conducted using paired two-way ANOVA with a Tukey post-hoc test or paired two-tailed t-test. Significant differences are indicated by P < 0.05. * Indicates difference between AIR + RV and AIR, # = CS + RV vs CS, and & = CS + RV vs AIR + RV. **C** Heat map of interferon response gene set in ALI-PBEC at 6hpi is also shown in a separate bar graph, in which the intensity of combined gene expression is shown as average Z scores. The Z scores for individual genes are represented in green and red, while the average Z scores (underneath the heat maps) of all genes in the gene set are represented in blue and red. The Z scores shown are relative to the average gene expression of the corresponding dataset at that time point. Below the heat maps, the average Z scores in blue represent downregulated gene expression while red color shows upregulated gene expression. **D** ALI-PBEC were exposed to CS or air control and then directly infected with RV-A16 (MOI 1) for 1 h and cultured in presence or absence of IFN-β or IFN-λ1 for 24 h. The levels of RV-A16 vRNA at 24hpi was measured by qPCR. Data are shown as target gene expression normalized for *RPL13A* and *ATP5B*. Data are mean values ± SEM; n = 4 different donors. Analysis of differences was conducted using paired two-way ANOVA with a Tukey post-hoc test. Significant differences are indicated by *P < 0.05

To determine the effect of CS exposure on viral replication, we exposed ALI-PBEC derived from COPD and non-COPD donors to CS followed by RV-A16 infection. The experimental design is summarized in Additional file 1: Fig. S1. At 24 and 48 h post infection (hpi), intracellular viral RNA (vRNA) levels of RV-A16 were significantly increased in CS-exposed cultures when compared to air-exposed cultures (Fig. 1A and 1B). This effect was similar between COPD (red dots) and non-COPD donors (black dots) (Fig. 1B). Further confirmation was obtained by measuring RV-A16 infectious particles in the apical wash at 24hpi (Additional file 1: Fig. S2A). To explore mechanisms associated with enhanced viral replication in ALI-PBEC after CS exposure, we performed bulk RNA-Seq. We initially compared gene expression of ALI-PBEC exposed to CS, RV-A16 or this combination with air-exposed controls for COPD and non-COPD donors separately. Comparison of these different exposures to air controls revealed a higher number of differentially expressed genes (DEGs) in ALI-PBEC from COPD donor cultures compared to non-COPD donors at 24hpi (Additional file 1: Table S1). The Venn diagram depicts overlapping and unique DEGs comparing CS exposure, RV16 infection and CS combined with RV16 infection compared to air controls, for COPD and non-COPD donors separately (Additional file 1: Fig. S2B). We noted that, the response of ALI-PBEC to CS and RV-A16 exposures was not significantly different between COPD and non-COPD donors (principle component analysis [PCA]; Additional file 1: Fig. S2C). Therefore, we combined both these groups in all the following analyses to increase power of the comparisons for the exposures.

When we explored the associated mechanisms via RNA-Seq analysis, we focused first on early effects of CS exposure that may have contributed to the later detected increase in RV-A16 virus levels. CS exposure significantly downregulated expression related to interferon (IFN) response genes at 6hpi (Fig. 1C), which was further confirmed by measuring expression of the IFN-response gene *RSAD2* by qPCR (Additional file 1: Fig. S2D). In addition, CS exposure significantly decreased expression of pattern recognition receptors (PRRs) involved in viral recognition, including membrane-bound toll like receptor 3 (TLR3), cytoplasmic retinoic acid-inducible gene (RIG-I, gene name *DDX58*) and melanoma differentiation-associated protein 5 (MDA5, gene name *IFIH1*) (Additional file 1: Fig. S2E). Since CS exposure decreased IFN responses at an early time-point and increased viral levels at later timepoints, we evaluated if addition of type I and III IFNs ALI-PBEC cultures could normalize RV-A16 level in CS-exposed cultures. Viral levels in CS-exposed ALI-PBEC at 24hpi was normalized to air-exposed culture levels in the presence of recombinant IFN-β and -λ1, indicating that presence of both epithelial type I and III IFNs are important for reducing RV-A16 levels after CS exposure (Fig. 1D). Together, these results demonstrate that CS exposure attenuates the interferon response at an early phase after smoke exposure, and that addition of interferons limits the increased in viral infection upon CS exposure.

### Cigarette smoke exposure alters rhinovirus-induced antiviral responses and enhances inflammatory mediator production

**Fig. 2:**
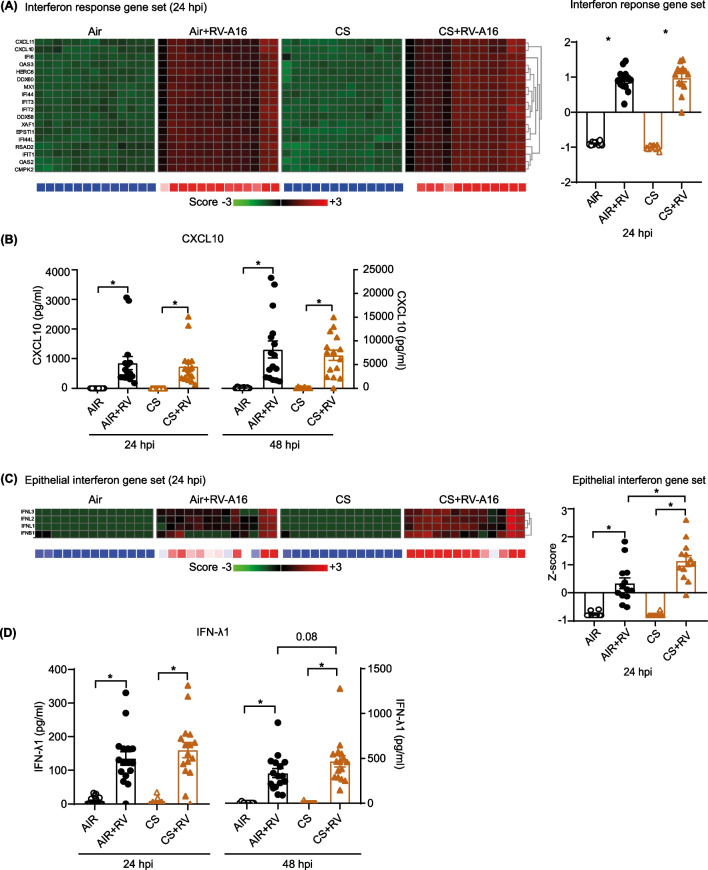

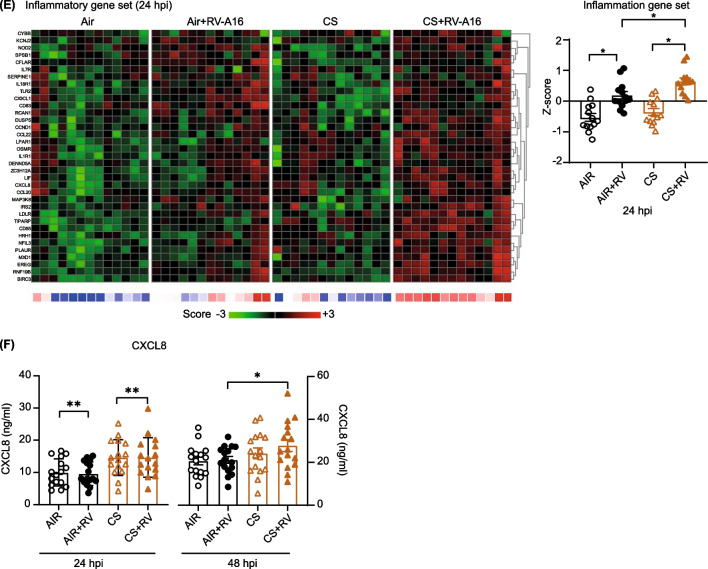
The modulation of antiviral responses and enhancement of rhinovirus-induced inflammatory responses by cigarette smoke. **A**, **C**, **E** Heat maps showing interferon response gene set, epithelial interferon gene set and inflammatory gene set in ALI-PBEC at 24hpi. For all heatmaps in this figure, the intensity of combined gene expression is shown as Z scores. Below the heat maps, blue represents downregulated gene expression while red color shows upregulated gene expression. The Z scores in all heatmaps for individual genes are represented in green and red, while the average Z scores (underneath the heatmaps) of all genes in the gene set are represented in blue and red. The Z scores shown are relative to the average gene expression of the corresponding dataset at that time point. Data are mean values ± SEM. n = 13 different donors. Analysis of differences was conducted using paired one-way ANOVA with a Tukey post-hoc test. Significant differences are indicated by *P < 0.05. **B**, **D**, **F** Protein levels of CXCL10, IFN-λ1 and CXCL8 were measured by ELISA. Data are mean values ± SEM; n = 16 different donors. Analysis of differences was conducted using paired one-way ANOVA or two-way ANOVA with a Tukey post-hoc test. Significant differences are indicated by *P < 0.05

Next, we explored if CS exposure continued to suppress antiviral responses also at later time-points. Expression of the IFN response gene set at 24hpi was similar between CS and air-exposed cell cultures (Fig. 2A), despite the higher levels of virus in the CS-exposed cultures at this point, suggesting a continued suppression. RV-A16 infection at this time-point, markedly induced expression of this gene set. When we verified protein levels of CXCL-10, (an interferon-induced cytokine) that is part of this gene set, indeed RV-A16-exposed cell cultures had increased levels at 24hpi and 48hpi in the basal medium, while levels between air-exposed and CS-exposed cell cultures were similar (Fig. 2B). Interestingly, RV-A16 furthermore significantly increased expression of epithelial-related IFN genes at 24hpi, which was further increased after CS exposure (Fig. 2C), likely related to the level of virus infection (Fig. 1A). We further analyzed the kinetics of the expression of *IFNB1* and *IFNL1* by qPCR (Additional file 1: Fig. S3A) and of IFN-λ1 protein levels (Fig. 2D) at 6, 24 and 48hpi. Particularly *IFNL1* expression showed comparable kinetics to the virus level kinetics in Fig. 1A. Together these data show that at later timepoints CS no longer alters RV-A16-induced interferon response genes, while RV-A16, especially in combination with CS, increases expression of epithelial interferons.

Using gene set enrichment analysis (GSEA) and ingenuity pathway analysis (IPA), we identified (and selected) gene sets based on their differential expression following RV-A16 exposure at 24hpi (Additional file 1: Table S2). In addition, we used network analysis from IPA to identify pathways linked with the interferon response (Additional file 1: Fig. S3B). From this analysis, pathways related to inflammation, necroptosis and complement were identified and further analyzed. A set of inflammation-related genes, including *CXCL8*, was significantly increased after RV-A16 infection and even further (significantly) when combined with CS exposure compared to either exposures alone (Fig. 2E). Also on protein level, at 48hpi significantly more CXCL8 was detected in cultures with combined exposure of CS and RV-A16 compared to CS or RV-A16 exposure alone (Fig. 2F).

Besides inflammation, we furthermore observed that RV-A16 infection alone mainly increased expression of genes associated with necroptosis (Additional file 1: Fig. S4A and S4B), whereas expression of genes associated with autophagy were upregulated by CS exposure in ALI-PBEC from all donors at 6 h (Additional file 1: Fig. S4C and Fig. S4D). Since these are various forms of cell death, we evaluated if the plasma membrane was damaged, a key feature of cells undergoing various types of cell death, using an LDH assay. Levels of LDH were minimal, but there was a limited, significant increase in LDH in CS-exposed ALI-PBEC at 24 h and a further increase was found in CS and RV-A16 exposed cells at 48hpi (Additional file 1: Fig. S4E). Finally, we also observed that RV-A16 at 24hpi induced a significant increase in expression of a set of complement-related genes (Additional file 1: Fig. S4F and Fig. S4G). Overall, these results demonstrate that at later timepoints, combined exposure of CS and RV-A16 promotes expression of inflammation-related genes and proteins and RV-A16 alone furthermore affects pathways related to necroptosis and complement.

### Role of oxidative stress and growth differentiation factor 15 in the increased viral infection following cigarette smoke exposure

**Fig. 3:**
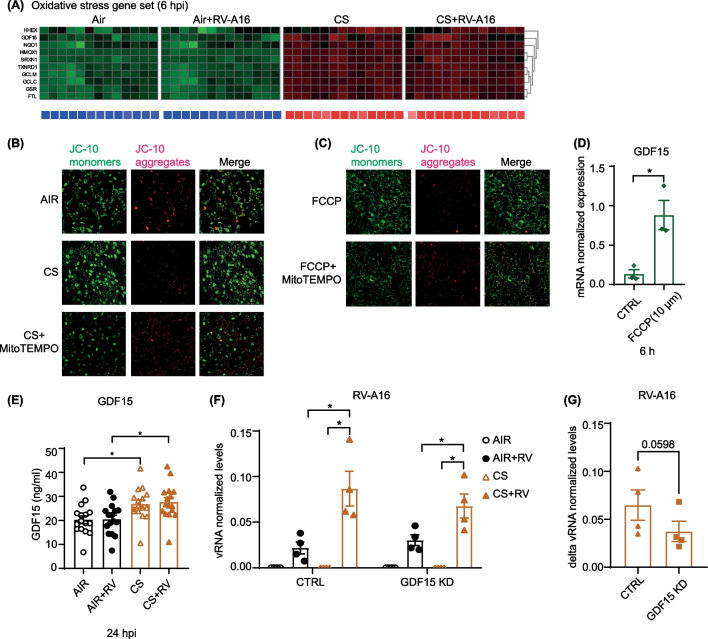
Enhancement of rhinovirus infection by cigarette smoke-induced oxidative stress and growth factor differentiation 15. **A** Heatmap of oxidative stress gene set was shown. Below the heatmaps, blue represents downregulated gene expression while red color shows upregulated gene expression. The Z scores in the heatmap for individual genes are represented in green and red, while the average Z scores (underneath the heatmaps) of all genes in the gene set are represented in blue and red. The Z scores shown are relative to the average gene expression of the corresponding dataset at that time point. **B**, **C** The mitochondrial membrane potential after CS exposure or FCCP treatment with/without mitoTEMPO was measured by JC-10 assay. Green color shows JC-10 monomers while red color is JC-10 aggregates. **D** The expression of GDF15 after FCCP treatment was measured by qPCR at 6 h. Data are mean values ± SEM. n = 3 different donors. Analysis of differences was conducted using paired two-tailed t test. Significant differences are indicated by *P < 0.05. **E** Protein levels of GDF15 after CS and RV-A16 exposure were measured by ELISA. Data are mean values ± SEM. n = 16 different donors. Analysis of differences was conducted using paired one-way ANOVA with a Tukey post-hoc test. Significant differences are indicated by *P < 0.05. **F**, **G** CS and RV-A16 exposure were done in *GDF15* knockdown cells and controls and incubated for 24 h after washing apically with PBS. The levels of RV-A16 vRNA at 24hpi was measured by qPCR. Data are shown as either target gene expression normalized for *RPL13A* and *ATP5B* or delta expression between CS + RV and Air + RV groups in control and *GDF15* knockdown cells. Data are mean values ± SEM; n = 4 different donors. Analysis of differences was conducted using paired two-way ANOVA with a Tukey post-hoc test or paired two-tailed t test. Significant differences are indicated by *P < 0.05

In addition to antiviral defenses, we further explored other early transcriptional responses of ALI-PBEC to CS exposure and how they are connected to increased viral levels at later time-points. Based on the results of our GSEA and IPA pathway analysis (Additional file 1: Tables S3 and S4), we focused on responses related to oxidative stress and autophagy. CS exposure significantly increased the expression of genes associated with oxidative stress in ALI-PBEC from all donors, which includes an increased expression of growth differentiation factor-15 (*GDF15)* (Fig. 3A). We confirmed this by measuring *HMOX-1*, a marker gene for oxidative stress, by qPCR, which showed a significant increase upon CS exposure at 6 h (Additional file 1: Fig. S5A).

Reactive oxygen species can contribute to mitochondrial damage and thereby impair antiviral signaling, as mitochondria plays a central role in antiviral signaling by mitochondrial antiviral signaling protein (MAVS) [14]. We therefore first assessed the effect of CS on mitochondrial membrane potential (MMP) by performing a JC-10 assay. JC-10 monomers (in green) were increased 2 h after CS exposure and JC-10 aggregates (in red) were decreased, indicating reduced MMP (Fig. 3B); similar findings were obtained for the positive control, FCCP, that promotes a decrease in the MMP (Additional file 1: Fig. S5B). Next, we pre-treated ALI-PBEC with mitoTEMPO to study whether this mitochondria-targeted anti-oxidant can reverse the CS-induced decrease in MMP. Interestingly, pre-treatment of ALI-PBEC with mitoTEMPO abolished the effects of CS (Fig. 3B) and FCCP (Fig. 3C) on MMP. These observations indicate that CS cause mitochondrial damage by decreasing MMP, and that this is mediated in part by the ability of CS to increase oxidant production by mitochondria.

We next investigated the potential consequences of this CS-induced decrease in MMP. We observed that at 6 h FCCP treatment increased expression of *GDF15,* a gene in the oxidative stress gene set, suggesting a possible association of GDF15 and oxidative stress-mediated decrease in MMP and viral clearance (Fig. 3D). Since GDF15 was previously demonstrated to alter antiviral responses in human airway epithelium [15], we therefore next explored the association between the CS-induced increase in viral replication and GDF15 production. We observed that CS not only increased *GDF15* gene expression, but also caused a significant increase in GDF15 protein levels in the basal medium at 24hpi post CS exposure (Fig. 3E).

To investigate if *GDF15* is indeed connected to the increased viral levels after CS-exposure, we next optimized *GDF15* gene knockdown in our cultures. GDF15 knockdown was achieved in submerged PBEC using non-plasmid based delivery of CRISPR-Cas9 by electroporation and lipofectamine, which is expected to have less off target effects when compared to knockdown using lentiviral transfection. Initial transfection efficiency in submerged PBEC was ~ 95% based on the number of transfected cells and target efficiency after prolonged differentiation was ~ 40% as assessed by measuring band intensities (Additional file 1: Fig. S5C). As a result, baseline protein secretion of GDF15 was significantly decreased to around 50% in differentiated *GDF15* knockdown cultures compared to controls (Additional file 1: Fig. S5D). Also, the increase in GDF15 protein levels after CS exposure was lower in *GDF15* knockdown cultures compared to controls (Additional file 1: Fig. S5E). RV-A16 replication was increased after CS exposure in both control (no *GDF15* knockdown) and *GDF15* knockdown cultures compared to air-exposed cells at 24hpi (Fig. 3F). Whereas this increase was lower for all four donors in *GDF15* gene-edited ALI-PBEC compared to controls, this difference did not reach statistical significance (Fig. 3G). Together with the cigarette smoke-induced increase in expression and secretion of GDF15, these data suggest involvement of GDF15 in the observed increase in viral replication after CS exposure.

### Cigarette smoke exposure alters cellular metabolism and enhances l-lactate, leading to enhanced rhinovirus infection

**Fig. 4:**
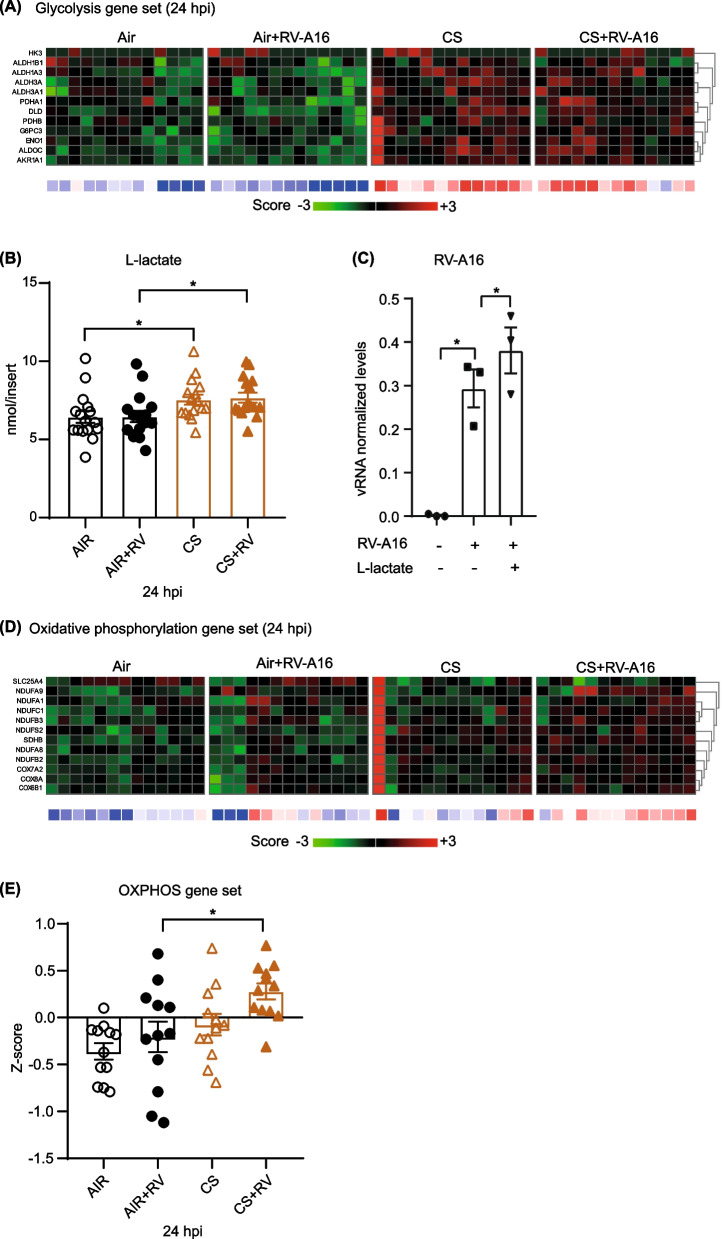
The mediation of CS-induced increase in RV-A16 infection via l-lactate production. ALI-PBEC were exposed to CS or air control and then directly infected with RV-A16 (MOI 1) for 1 h and were incubated for 24 h. **A**, **D** Heat maps showing glycolysis gene set and oxidative phosphorylation gene set. Below the heat maps, blue represents downregulated gene expression while red color shows upregulated gene expression. The Z scores in all heatmaps for individual genes are represented in green and red, while the average Z scores (underneath the heat maps) of all genes in the gene set are represented in blue and red. The Z scores shown are relative to the average gene expression of the corresponding dataset at that time point. **B** The release of l-lactate at 24hpi was measured. Data are mean values ± SEM. n = 16 different donors. **C** ALI-PBEC pre-treated with l-lactate were infected with RV-A16 (MOI 1) for 1 h and collected at 24hpi. The levels of RV-A16 vRNA at 24hpi was measured by qPCR. Data are shown as either target gene expression normalized for *RPL13A* and *ATP5B*. Data are mean values ± SEM. n = 3 different donors. **E** The intensity of combined oxidative phosphorylation gene expression was shown as Z scores. Data are mean values ± SEM; n = 13 different donors. All the analysis of differences in this figure was conducted using paired one-way ANOVA with a Tukey post-hoc test. Significant differences are indicated by * P < 0.05

Besides effects on oxidative stress-related genes, CS exposure significantly increased gene expression associated with glycolysis in ALI-PBEC (Fig. 4A). Indeed, levels of l-lactate, a by-product of glycolysis, were significantly increased at 24 h after CS exposure (Fig. 4B). To investigate if the CS-induced increase in l-lactate production also affected viral replication in ALI-PBEC, ALI-PBEC were treated with l-lactate which resulted in significant higher viral replication compared to untreated RV-A16-infected controls (Fig. 4C). In addition, at 24hpi there was elevated expression of oxidative phosphorylation genes in ALI-PBEC exposed to a combination of CS and RV-A16 compared to the other groups (Fig. 4D and E). In summary, CS exposure increases expression of glycolysis-related gene set and l-lactate, a by-product of glycolysis, which further contributes to enhanced viral replication.

### Deconvolution analysis predicts a decrease in ciliated cells following cigarette smoke exposure

**Fig. 5:**
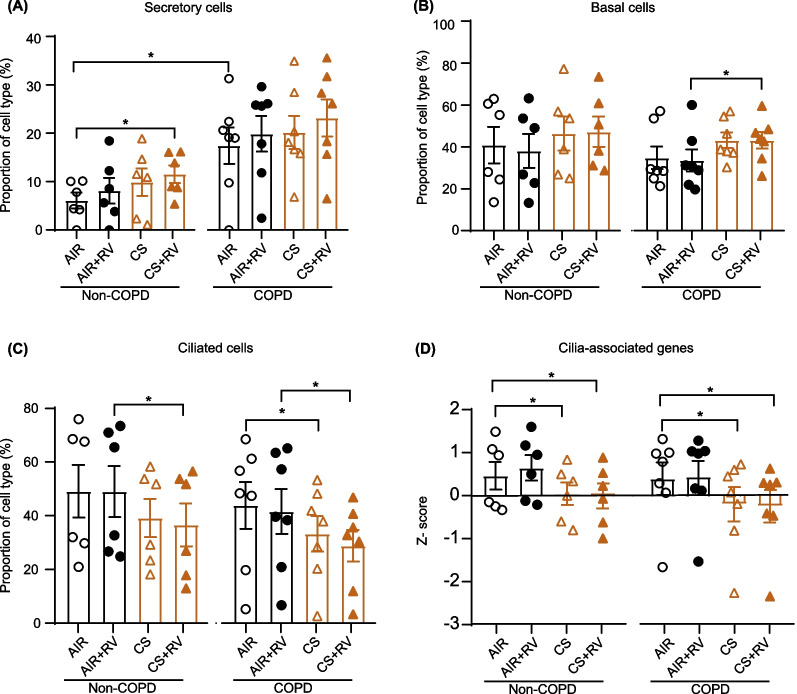
Relative proportion of specific cell types after cigarette smoke exposure and RV-A16 infection in primary human bronchial epithelial cell cultures. ALI-PBEC isolated from COPD and non-COPD donors were exposed to CS or air control and then directly infected with RV-A16 (MOI 1) for 1 h and were incubated for 24 h. The relative proportion of different cell types, secretory cells (**A**), basal cells (**B**) and ciliated cells (**C**) in ALI-PBEC after CS and RV-A16 exposure as predicted by cellular deconvolution of the transcriptomic datasets. **D** Z scores of differentially expressed gene sets related to cilia-associated genes are shown at 24hpi. Data are shown as mean ± SEM; n = 13 different donors. Analysis of differences was conducted using paired two-way ANOVA with a Tukey post-hoc test or unpaired two-tailed t test. Significant differences are indicated by *P < 0.05

Previously, we showed that RV mainly infects ciliated cells in ALI-PBEC [16]. Therefore, we next studied whether the impact of CS on RV infection could additionally be related to changes in cellular composition. We therefore used cellular deconvolution analysis to estimate basal, secretory, ciliated and rare epithelial cell types in ALI-PBEC at baseline and changes after CS and/or RV-A16 exposures in COPD and non-COPD donors at 24hpi (Additional file 1: Fig. S6A). At baseline, we observed a significant higher proportion of secretory cells in ALI-PBEC from COPD donors compared to non-COPD donors (Fig. 5A). CS exposure significantly increased expression of genes associated with basal cells in ALI-PBEC from COPD donors while this was not observed in non-COPD donors (Fig. 5B). Genes associated with ciliated cells in ALI-PBEC were significantly lower expressed after CS exposure in both COPD and non-COPD donors (Fig. 5C). In line with this, expression of genes associated with cilia (e.g. dyneins) were also significantly decreased after CS exposure (Fig. 5D and Additional file 1: Fig. S6B). Overall, deconvolution analysis predicted a decrease in proportion of ciliated cells after CS exposure, and a concomitant CS-induced decrease in expression of genes related to cilia in ALI-PBEC from both COPD and non-COPD donors, which may impact viral infection.

### In vivo responses of nasal epithelium to experimental rhinovirus infection are comparable to in vitro responses

**Fig. 6:**
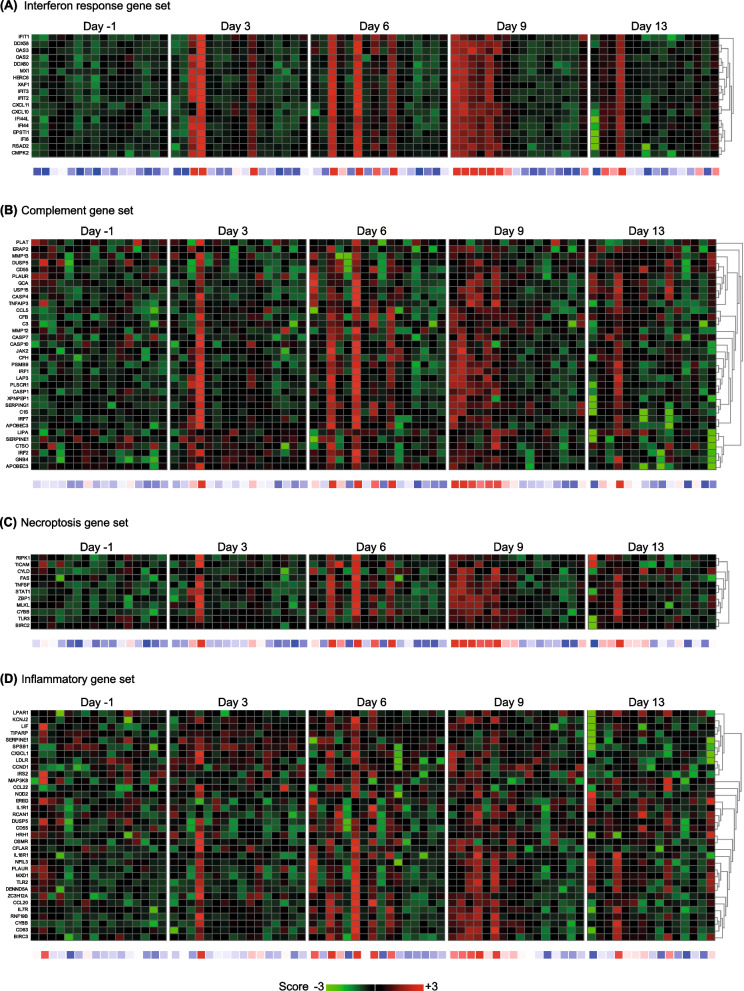
Effects of RV-A16 infection on transcriptional responses of the nasal epithelium in vivo. Nasal brushings were obtained from non-COPD donors on day 3, 6, 9 and 13 after RV-A16 exposure and 1 day before as baseline. RNA was isolated and analyzed using RNA-Seq in a similar analysis platform as for the cultured ALI-PBEC. **A**–**D** The heatmaps of interferon response gene set, complement gene set, necroptosis and inflammatory gene sets are shown and the intensity of combined gene expression is shown as Z scores. Below the heatmaps, blue color depicts downregulated and red color depicts upregulated expression; n = 16 different non-COPD donors. The Z scores in all heatmaps for individual genes are represented in green and red, while the average Z scores (underneath the heatmaps) of all genes in the gene set are represented in blue and red. The Z scores shown are relative to the average gene expression of the corresponding dataset at that time point

To relate our in vitro findings to those inhuman subjects, we compared transcriptional responses of ALI-PBEC to RV-A16 infection with those measured in nasal brushings after intranasal RV-A16 challenge of human subjects [13]. We selected the IFN response gene set from Fig. 1B and tested if these genes were also affected after in vivo RV-A16 challenge. The IFN response gene set was increased in 10 of the 16 included individuals between day 3 to day 9 after intra-nasal exposure to RV-A16, with a more profound increase at day 9 (Fig. 6A). Interestingly, individuals with high IFN responses also displayed a similar enhanced expression of previously tested gene sets related to the complement system (Fig. 6B), necroptosis (Fig. 6C) and inflammation (Fig. 6D). The Z scores of interferon response genes set strongly correlated with the Z scores of gene sets related to complement, necroptosis and inflammation (Additional file 1: Fig. S7A–C). The correlation analysis shows that individuals with high gene expression of the interferon response gene sets also had high expression of genes related to complement, necroptosis and inflammation in airway epithelium at different time points post-RV-A16 exposure. These results indicate that RV-A16 infection promotes comparable transcriptional responses in the airway epithelium in our in vitro ALI-PBEC model and in vivo, in a selected group of RV-responsive individuals.

## Discussion

In this study, we investigated mechanisms underlying the increased RV-A16 infection upon CS exposure of ALI-PBEC using a transcriptomics-based approach combined with functional studies. In line with various studies in other epithelial cell models, we confirm that whole CS exposure increased rhinovirus load in ALI-PBEC cultures. Importantly, we identified an impairment in expression of interferon response genes early after CS exposure, likely contributing to increased rhinovirus replication based on the ability of added interferons to reverse the effects of CS exposure on viral infection. Using functional studies, we additionally demonstrated that enhancement of GDF15 and l-lactate production after CS exposure contributes to increased viral replication. At a later phase, despite higher viral replication in CS-exposed ALI-PBEC, interferon response genes remained unaltered, while expression of epithelial interferons and inflammatory mediators were increased. Finally, analysis of data from a human experimental RV-A16 challenge study demonstrated that our results in vitro show overlap with similar pathways after in vivo RV-A16 exposure, including interferon response, complement, necroptosis and inflammation.

CS exposure of ALI-PBEC decreased expression of IFN response genes and PRRs implicated in viral recognition at 6hpi, which may have hampered antiviral defenses in the early stages of viral infection and thereby contributed to the observed increase in viral replication at 24 and 48hpi. One study demonstrated that early administration of IFN-β protects CS-exposed mice from lethal influenza virus infection [17]. In ALI-PBEC, we showed that pre-treatment with either IFN-β or IFN-λ1 decreased RV-A16 replication after CS exposure, confirming their protective effect in an early phase. At 24hpi, RV increased expression of IFN response genes and CXCL10 production in ALI-PBEC. Another study showed that RV infection of bronchial epithelium leads to a virus titer-dependent expression of interferon-response genes such as *CXCL10* and *RSAD2* [18]. Despite higher viral replication in CS-exposed epithelial cultures at 24hpi (which would be expected to trigger higher levels of CXCL10 and expression of interferon response genes), expression of these interferon response genes and CXCL10 protein levels were similar to infected cultures without CS exposure, indicative of a dampened interferon response. In contrast to these interferon response genes, expression of interferon genes in RV-A16 infected cells was increased by CS exposure at 24hpi. This effect likely relates to the higher viral load after CS exposure. Even though epithelial interferon production was increased at 24hpi, the viral replication was not decreased in CS-exposed ALI-PBEC at 48hpi, which may be related to the observation that interferon-response genes were not altered by CS exposure despite the higher viral load and higher interferon production. Rather, CS exposure increased inflammation and necroptosis and therefore increased interferon could potentially contribute to excessive lung inflammation and tissue damage at a later stage [19]. Altogether, our studies indicate that there might be distinct effects of smoke on antiviral responses between early and late stages of RV infection.

We further identified CS-mediated mechanisms that contribute to rhinovirus infection and showed that CS exposure increased oxidative stress, which may contribute to mitochondrial damage [20]. We hypothesize that this damage is responsible for the increase in the stress-induced cytokine/growth factor GDF15, that we found to directly contribute to impaired viral clearance. This hypothesis is supported by a previous study showing that a decrease in MMP impaired MAVS-mediated antiviral signaling in the mitochondria, leading to higher viral infection [21]. We showed that CS exposure and FCCP (positive control for depolarization of MMP) both increased expression of GDF15 in ALI-PBEC. GDF15 levels are elevated in COPD patients [22] and its epithelial expression is induced by CS [23]. A link between GDF15 and viral replication was supported by a study in which *GDF15*-overexpressing mice and GDF15-treated bronchial epithelial cells showed higher viral replication, which was attributed to an inhibition of IFN-λ1 [15]. However, whether GDF15 contributes to the increased RV replication following CS exposure in ALI-PBEC was unknown. We demonstrated that CS increased both RV-A16 vRNA and GDF15 levels at 24hpi, and that IFN-λ1 levels were similar upon RVA-16 infection in both air and CS-exposed cells. Whereas we observed that partial knockdown of *GDF15* appeared to decrease the effect of CS exposure on RV replication, this was based on a limited number of donors and the differences did not reach statistical significance. Nevertheless, our data do not allow us to conclude that GDF15 mediates increased RVA-16 replication by decreasing IFN-λ1 expression, as IFN-λ1 expression is regulated by RV-A16 vRNA levels (which are increased upon smoke exposure). Future studies using e.g. CS exposure and polyI:C stimulation instead of RV-A16 infection to increase IFN-λ1 expression could shed more light on this, but are beyond the scope of the present study. Collectively, these findings suggest that GDF15, increased by CS directly by oxidative stress or indirectly by mitochondrial membrane depolarization, may contribute to the CS-induced increase in RV-A16 infection in ALI-PBEC.

Next, we investigated changes in transcriptional responses by CS at 24hpi and showed increased expression of a glycolysis-related gene set. A recent study showed that lactate derived from glycolysis was found to target the MAVS protein and thus inhibits IFN production and enhances viral replication in mice [24]. In line, we showed that pretreatment of ALI-PBEC with sodium l-lactate, which we found to be increased upon CS exposure, enhanced RV-A16 replication. Apart from glycolysis, we also demonstrated that oxidative phosphorylation was only enhanced by the combination of both CS and RVA-16 exposure. Therefore, we illustrate that CS exposure changes the glycolysis metabolic pathway, which further contributes to viral infection.

In our study, there was no significant difference in response of the exposures between COPD and non-COPD-derived cultures. We included cells derived from moderate COPD patients (Stage II) and not severe COPD patients (Stage III or IV) and have compared COPD and non-COPD using 8 donors in each group, which may have lacked sufficient power to reach statistical significance as some loss of phenotype in culture is to be expected. Some phenotype is however still present as deconvolution analysis showed a higher proportion of secretory cells at baseline in cultures from COPD donors compared to non-COPD controls. In COPD patients, immunohistochemistry staining of the large airways showed increased MUC5AC levels, a marker for goblet cells [25]. Thereby, in line with our previous findings [26], the ALI-PBEC cultures from COPD patients did indeed retain certain intrinsic features in culture. Furthermore, our analysis shows that CS exposure reduces the proportion of ciliated cells and cilia-associated gene expression, which may contribute to impaired mucociliary clearance [27]. The deconvolution analyses are based on a short follow-up after CS exposure (up to 24 h), and therefore indicates predicted changes in cell proportion in ALI-PBEC at a later phase, rather than definitive alterations in the percentage of cell numbers. Therefore, it would furthermore be valuable to also investigate the effect of chronic CS exposure of the epithelium on viral infection for a closer relevance to COPD [9, 28]. The results of this study do not provide evidence for a role of interactions among the pathways related to interferons, glycolysis and GDF15 in mediating the effect of CS on RV-A16 replication, but more studies are required for firm conclusion regarding such interactions. Furthermore, our model is limited because it only comprises (differentiated) epithelial cells, and the role of immune cells, bronchial smooth muscle cells (and other structural cells) was not investigated. RV-A16 can drive the immune response against viral replication by infecting bronchial epithelium or macrophages [29] or bronchial smooth muscle cells [30] directly. Use of co-culture models or ex vivo precision-cut lung slices, would be valuable to study cell interactions.

Overall, our study provides evidence for a functional involvement of early and late anti-viral responses, along with GDF15 and lactate production in CS-mediated increase in RV-A16 infection. These findings aid in unravelling the complexity related to this increased infectivity and may promote research into therapeutically targeting of these pathways for virus-induced COPD exacerbations.

### Supplementary Information

**Additional file 1.** Supplementary materials.

## Data Availability

All data needed to evaluate the conclusions in the paper are present in the paper and/or the Additional file 1. RNA-sequencing datasets will be uploaded in the online repository. Additional data related to this paper may be requested from the authors.
