# Supplementary material for: Acute cigarette smoke exposure leads to higher viral infection in human bronchial epithelial cultures by altering interferon, glycolysis and GDF15-related pathways
Source: Respir Res. 2023 Aug 23;24:207. doi: 10.1186/s12931-023-02511-5 (PMC10464373; doi:10.1186/s12931-023-02511-5)
Supplement: Supplementary file 1 — Additional file 1. Supplementary materials. [file 12931_2023_2511_MOESM1_ESM.docx]

Additional file for

**Acute cigarette smoke exposure leads to higher viral infection in human bronchial epithelial cultures by altering interferon, glycolysis and GDF15-related pathways**

Ying Wang^1^, Dennis K. Ninaber^1^, Alen Faiz^2^, Abraham C. van der Linden^1^, Annemarie van Schadewijk^1^, René Lutter^3^, Pieter S. Hiemstra^1^, Anne M. van der Does^1^*, Abilash Ravi^1^*†

^1^Department of Pulmonology, Leiden University Medical Center, Leiden, the Netherlands

^2^University of Technology Sydney, Respiratory Bioinformatics and Molecular Biology (RBMB), School of Life Sciences, Sydney, Australia

^3^Department of Respiratory Medicine, Amsterdam University Medical Center, University of Amsterdam, Amsterdam, the Netherlands

*Shared senior authorship

†Address correspondence to:

Abilash Ravi, PhD

Department of Pulmonology, Leiden University Medical Center, the Netherlands

e-mail: a.ravi@lumc.nl

**This PDF file includes**:

Supplementary methods

Fig. S1-S6

Table S1-S5

**Supplementary Methods**

**Cell isolation and culture**

PBEC were isolated by scraping bronchial rings after incubation with protease XIV solution (Sigma-Aldrich, St. Louis, MO, USA) for 2 h. These cells were seeded and expanded in 6-well plates (P0, Corning Costar, Cambridge, USA) as described ^1^, until ~90% confluent after which cells were stored in liquid nitrogen upon trypsinization. When experiments were performed, thawed cells were first expanded in T75 flasks (Greiner Bio-One, Alphen a/d Rijn, the Netherlands). Then cells were transferred to 12-well Transwells (Corning Costar, Cambridge, USA) and differentiated at air-liquid interface as previously described ^2^. ALI-PBEC used were at passage 2.

**Cigarette smoke exposure:**

The apical surface of ALI-PBEC was washed with PBS 4h before the exposure to remove mucus. Well-differentiated cells in 12-well inserts were placed in either an air or whole CS exposure chamber. Whole CS was prepared by burning one cigarette (3R4F reference cigarettes ; University of Kentucky, Lexington, KY, USA) and subsequently infusing the whole cigarette smoke into the CS chamber while room air was delivered into the control exposure chamber for 4-5 minutes. Next, residual smoke in the chamber was removed for 10 minutes by infusing the chambers with air derived from the incubator. The weight of the outlet filter was measured before and after CS exposure to calculate the total weight of CS particles.

**RV-A16 stock preparation**

RV-A16 was obtained from ATCC (VR-283™; Wesel, Germany) and replicated in H1-HeLa cells (ATCC CRL-1958™). RV-A16 was collected and partially purified from H1-HeLa cells according to a previously published protocol ^2^. Infectious viral titers were determined by 50% tissue culture infective dose (TCID50) measurements.

**TCID50 measurements**

Serial dilutions of apical washes were retrieved by apically adding 200 μl prewarmed PBS for 10 minutes. The collected washes were used to infect H1-HeLa cells in DMEM medium (Stemcell Technologies, Germany) with 4% FBS (Bodinco, the Netherlands) for 5 days. Live and dead cells visualized under the microscope were counted manually and TCID50 was calculated by the Spearman & Kärber algorithm ^3^.

***GDF15* knockdown**

This non-lentiviral based method was selected because it is less likely to result in off-target editing. We did not generate a clonal cell population and/or select successfully transfected cells in view of the limited life-span of primary cells, and therefore knockout of the *GDF15* gene was not achieved in all cells present in our cultures. Initially, we tested three predesigned Alt-R CRISPR-Cas9 guide RNA (crRNA; IDT): Hs.Cas9.GDF15.1.AA, Hs.Cas9.GDF15.1.AB, Hs.Cas9.GDF15.1.AC), of which we selected Hs.Cas9.GDF15.1.AA (sequence: TCCCACGACCTTGACGCCGTCGG) based on comparison of GDF15 secretion levels. Next the crRNA (Hs.Cas9.GDF15.1.AA or Alt-R CRISPR-Cas9 Negative Control crRNA #1) was mixed with transactivating CRISPR RNA (tracrRNA, ATTO^TM^ 550) by heating at 95°C for 5 minutes and letting it cool down again at room temperature. Then the mixture was stored at -20°C up to 3 months. When required, Cas9 HiFi enzyme was added to the above mixture by incubating at room temperature for 20 minutes to form the ribonucleoprotein (RNP) complex. PBEC at passage 2 were transfected with the RNP complexes using two delivery methods, electroporation followed by lipofectamine transfection. In brief, the submerged PBEC in T75 flasks (Greiner Bio-One) were trypsinized and the washed cell suspension was electroporated with the RNP complexes and electroporation enhancer to knockdown *GDF15*. The electroporation assay was performed according to the online protocol from IDT: “Alt-RCas9 System, delivery of ribonucleoprotein complexes into HEK-293 cells using the Lonza Nucleofector system”. The electroporated cells were directly seeded in T75 flasks and incubated for 2 days. Next, these cells were again trypsinized and the washed cell suspension was transfected with the RNP complexes and Lipofectamine RNAiMAX (ThermoFisher) based on the online protocol from IDT (Alt-R CRISPR-Cas9 System, cationic lipid delivery of CRISPR ribonucleoprotein complexes into mammalian cells). In brief, 100,000 electroporated cells on day 2 were seeded per 24-well insert (Corning Costar or cellQART made by Sabeu GmbH&Co.KG, Northeim, Germany), directly mixed with the RNP complex in the presence of Lipofectamine RNAiMAX (ThermoFisher) for 6h. Later, the apical mixture of the RNP complex and lipofectamine was replaced with fresh medium on the apical side. After cells had reached confluence in the insert, they were cultured at the air-liquid interface (ALI) for 2 weeks to induce cell differentiation.

For analyzing and quantifying transfected cells, the tracrRNA used in this study was linked with ATTO^TM^ 550 fluorescent dye which allowed to visualize transfected cells and monitor transfection efficiency. The transfected cells were visualized by immunofluorescence at 24h after electroporation and 6h after lipofectamine transfection to determine transfection efficiency, which was ~95% (Fig. S5c). In addition, we also assessed target efficiency based on DNA mutations in genomic DNA from ALI-PBEC after 2 weeks of cell differentiation. The genomic DNA was extracted using the Maxwell^®^ 16 cell DNA purification (Promega) according to the manufacturer’s protocol. On-target genome of *GDF15* was detected using the T7 endonuclease I (T7EI) mismatch cleavage assay (Alt-R Genome Editing Detection Kit, IDT). In brief, the targeted genomic region of extracted DNA was amplified using *GDF15* primers (Table S4) and T7EI was used to digest reannealed PCR products, by which, mismatched DNA heteroduplexes were cleaved. Targeting efficiency was assessed by measuring band intensities using 2% agarose gel electrophoresis and these were quantified in Image Lab software (Bio-Rad).

**RNA isolation, sequencing and analysis**

Total RNA from lysed cells was robotically extracted using the Maxwell® 16 simply RNA tissue kit (Promega, Leiden, the Netherlands). The mRNA was isolated from the total RNA using oligo-dT magnetic beads. After fragmentation of the mRNA, cDNA synthesis was performed which was used for ligation with sequencing adapters and PCR amplification. Fragment analyzer was used to determine the quality and yield after sample preparation which was in the expected distribution size of between 300 and 500 base pairs. The library preparation quality was evaluated by mapping the raw data to annotated genomic references. Clustering and DNA sequencing using the NovaSeq6000 were performed according to the manufacturer’s protocol. A concentration of 1.1 nM of DNA was used as input. Image analysis, base calling, and quality check were performed with Illumina data analysis pipeline RTA, version 2.4.11, and Bcl2fastq, version 17. Sequence reads were trimmed to remove possible adapter sequences using cutadapt v2.10. Presumed adapter sequences were removed from the read when the bases matched a sequence in the adapter sequence set (TruSeq adapters). The trimmed reads were mapped to the Homo_sapiens.GRCh37.75 reference genome using a short read aligner based on Burrows Wheeler Transform (Tophat v2.0.14). The frequency of the reads mapped on the transcript was determined as read counts. We used a lower limit of read counts of genes >10 for downstream analysis. The read counts were analyzed used DESeq2 software package to determine the differentially expressed genes (DEGs) when comparing two groups. The DEGs with a log2 fold change of >1.5 and an adjusted P value <0.05 (q value) were considered statistically significant. The DEseq2 method uses Benjamini-Hochberg correction as an adjustment for false discovery rates (q value). For the heat maps, log_2_ gene expression values were normalized with Z scores calculated by using the equation Z=*X* – (μ/σ), where *X* is the value of the individual sample, μ is the average of the row, and σ is the SD of the row. The gene clustering method used for the heat maps was based on k-means clustering. The combination of Z scores for all genes shown in heatmaps are represented below the heat maps in red and blue, with red indicating high expression and blue indicating low expression.

**Gene set and pathway analysis**

The gene sets were identified by Gene Set Enrichment Analysis (GSEA) in the online platform, accessed via the website [www.gsea-msigdb.org](http://www.gsea-msigdb.org/). These gene sets were identified using the differentially expressed genes (DEGs) with a q value <0.05. The gene identifiers based on Ensembl ID, were uploaded in the investigate gene sets column of the website, and the overlapping gene sets were determined. The gene sets identified were plotted as heat maps to visualize gene expression of all individual genes and donors. In addition to gene sets analyzed, we further analyzed the DEGs using IPA analysis software (<http://www.ingenuity.com>, QIAGEN, Redwood, CA, USA) to visualize and understand complex omics data and perform insightful data analysis and interpretation. The IPA output represented is based on the q value and the log2 fold change of the DEGs when comparing 2 groups. IPA analysis displays the most significantly affected pathways with graphical illustrations showing genes involved in the pathway, with DEGs in red for upregulated and green for downregulated genes. We used both GSEA and IPA analysis to define gene sets related to their respective pathways. Based on the results of the gene set enrichment analysis (GSEA) and ingenuity pathway analysis (IPA), we selected certain gene sets related to various antiviral pathways for further analysis based on their differential expression following CS and/or RV-A16 exposure (Table S2 and S3).

**Quantitative real-time PCR (qPCR)**

For qPCR, the relative standard curve method was used to calculate arbitrary gene expression using CFX Maestro software (Bio-Rad, Veenendaal, the Netherlands). In brief, cDNA or positive controls were mixed with IQ SYBR green supermix (Bio-Rad) and primers. Next, the thermal cycling protocol (95^o^C for 3 minutes, 40 cycles for 5 seconds at 95^o^C and 30 minutes at 63^o^C, a melt curve from 65^o^C to 95^o^C) was used. For analysis, the reference genes ATP synthase F1 subunit Beta (*ATP5B*) and Ribosomal Protein L13a (*RPL13A*) were identified by Genorm method out of 8 candidate reference genes (Genorm, Primer design, Southampton, UK), and were used to calculate the normalized gene expression. The primer pairs are provided in Table S5.

**Enzyme-Linked Immunosorbent Assays (ELISA)**

We used several ELISA kits in this study, named IL-29 (IFN-λ1) human uncoated ELISA Kit (ThermoFisher), human IP-10/CXCL10 ELISA (BD Biosciences, the Netherlands), human GDF15 DuoSet ELISA (R&D Systems) and human IL-8/CXCL8 DuoSet ELISA (R&D Systems). The measurements of these proteins were performed according to the manufacturer’s instructions. In brief, the Nunc MaxiSorp flat-bottom 96-well plates (ThermoFisher) used for ELISA were coated with capture antibodies overnight and next blocked with blocking buffer. Then diluted samples and standards were added in the plates after removal of blocking buffer using the washing buffer. After incubation for 2h at RT a wash step was followed by incubation with detection antibodies. After washing, the streptavidin conjugated to horseradish-peroxidase (Streptavidin^HRP^) solution was added into the plate for 30 min. followed by washing and addition of a solution with TMB (3, 3′, 5, 5′-tetramethylbenzidine) buffer, TMB substrate and H_2_O_2_. Finally, this reaction was stopped by adding 2.5 M H_2_SO_4_ and the OD values were measured at 450 nm.

**L-lactate measurements**

L-lactate present in basal medium was measured using the L-Lactate Assay Kit (Colorimetric/Fluorometric, Abcam, Cambridge, UK). Samples and standards were diluted in Lactate Assay Buffer and mixed with 50 µl Reaction Mix (Lactate Enzyme Mix, Probe and Lactate Assay Buffer) for 30 minutes in the dark. The absorbance at 570 nm was measured using a microplate reader (Bio-Rad). The relative standard curve was used to calculate the L-lactate levels.

**Lactate Dehydrogenase (LDH) assay**

LDH levels were assessed in the basal medium of ALI-PBEC collected immediately (the medium was not frozen prior to analysis) after CS and RV-A16 exposure or controls. Fresh B/D medium was used a negative control and 100% lysate (0.1% (w/v) Triton X-100/PBS-treated cells) were used as a positive control. The basal medium of ALI-PBEC, negative or positive controls were mixed with working solution of CytoTox 96 Reagent in a 96-well plate, followed by incubation for 30 minutes (in the dark) and reaction was stopped using a stop solution according to the manufacturer’s instructions (CytoTox 96 Non-Radioactive Cytotoxicity Assay, Promega). The absorbance was measured at 490 nm and percentage cytotoxicity were calculated as 100 x OD value of the sample / OD value of positive control.

**Supplementary Figures**


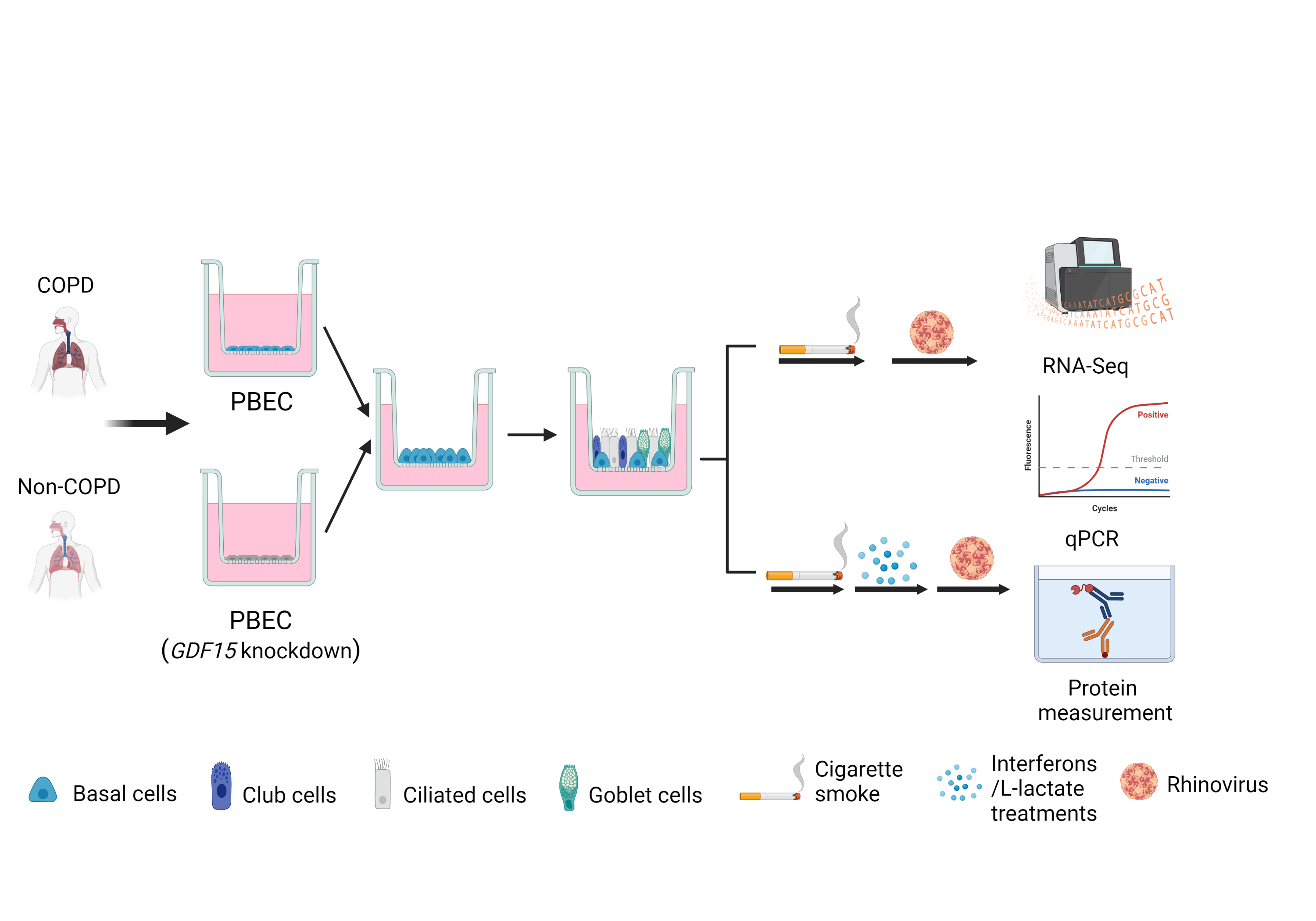


**Figure S1.** **Experimental design.** Primary human bronchial epithelial cells (PBEC) from both COPD and non-COPD donors were cultured on cell culture inserts. PBEC from 3 non-COPD donors were gene edited by CRISPR-Cas9 to achieve knock-down of *GDF15* expression followed by culture on inserts. Next apical medium was removed and cells were cultured at the air-liquid interface (ALI) and differentiated for 2 (*GDF15* knockdown and all treatments) or 4 weeks (RNA sequencing, RNA-Seq). Well-differentiated ALI-PBEC were exposed to CS followed by RV-A16 infection and harvested for RNA-seq, qPCR and protein measurements. For selected experiments, 10 ng/ml recombinant human IFN-β or IFN-λ1 was added after CS exposure, or cells were treated with 10 mM sodium L-lactate (not CS exposure), followed by RV-A16 infection. After 24h incubation, cells and basal medium were harvested for qPCR and protein measurements. Figure was created using BioRender.com.


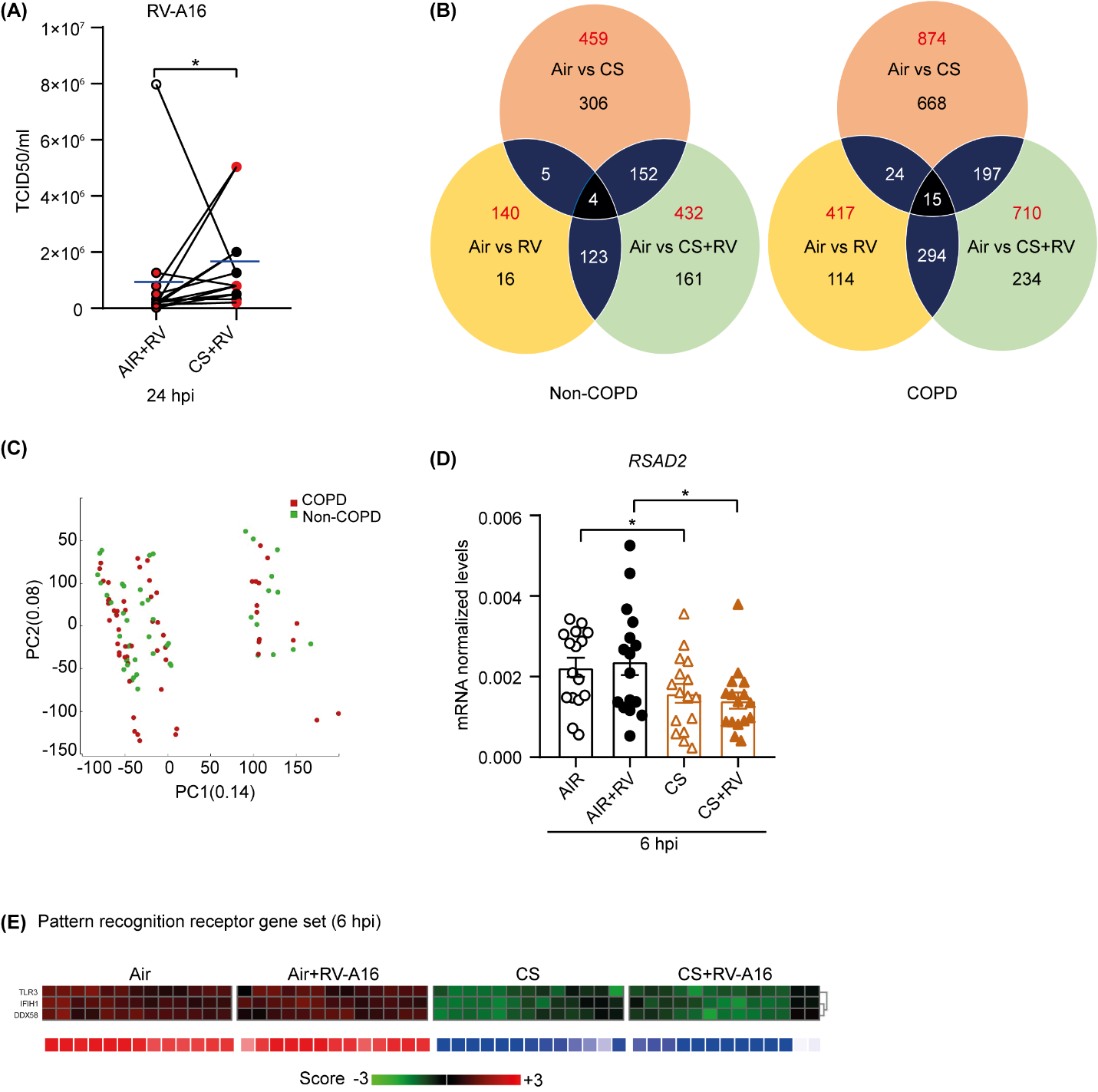


**Figure S2.** **Overview of differentially expressed genes and impact of cigarette smoke exposure on rhinovirus infection and antiviral defenses in differentiated primary human bronchial epithelial cells.**  ALI-PBEC were exposed to CS or air control and then directly infected with RV-A16 (MOI 1) for 1 h and incubated for 6 and 24hpi. **(A)** The expression of viral infectious particles was measured at 24hpi by TCID50 assay. Black symbols represent non-COPD donors, red symbols represent COPD donors. n*=*16 different donors. Data are shown as mean ±SEM. For statistical analysis, paired, two-tailed, t-test was used: *p<0.05. **(B)** Venn diagrams depicting the number and the overlap of DEGs between different comparisons. ALI-PBEC exposed to CS or RV-A16 or the combination was compared to air and mock infected controls. These comparisons are shown for COPD (n*=*7) and non-COPD (n*=*6) donors separately. **(C)** A principal component analysis (PCA) was performed comparing COPD and non-COPD donors based on RNA-Seq data. Green dots represent non-COPD donors, red dots represent COPD donors. **(D)** The expression of *RSAD2* was measured by qPCR at 6hpi. Black symbols represent non-COPD donors and red symbols represent COPD donors. n*=*16 different donors. Data are shown as mean ±SEM. For statistical analysis, paired one-way ANOVA with a Tukey post-hoc test was used: *p<0.05. **(E)** Heatmaps of pattern recognition receptor gene set in ALI-PBEC at 6hpi are shown. The intensity of combined gene expression is shown as Z scores. The Z scores for individual genes are represented in green and red, while the average Z scores (underneath the heat maps) of all genes in the gene set are represented in blue and red. The Z scores shown are relative to the average gene expression of the corresponding dataset at that time point. Below the heat maps, blue represents downregulated gene expression while red color shows upregulated gene expression. n=13 different donors.

**
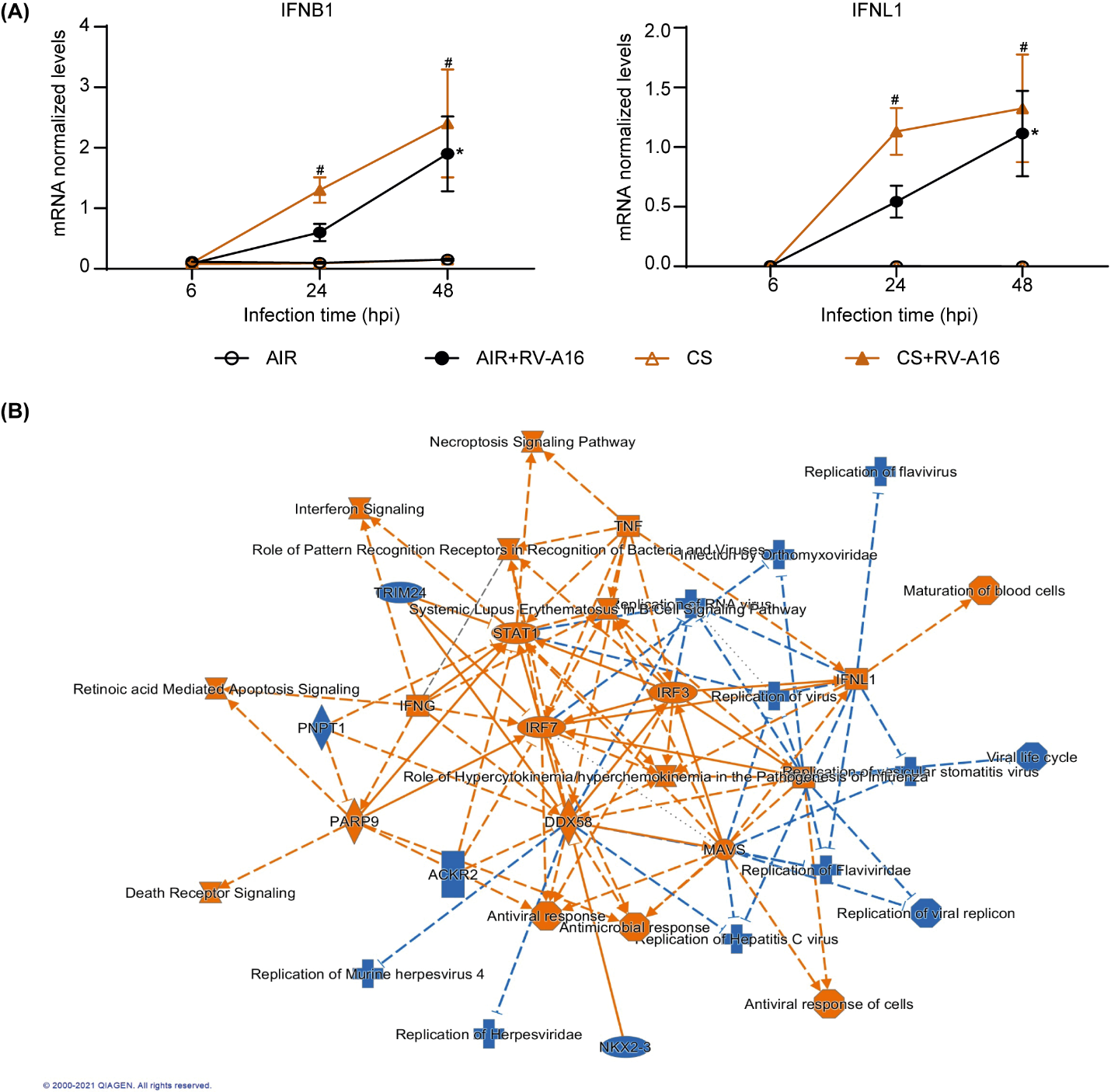
**

**Figure S3. Gene expression of interferons and pathways linking to interferon expression in primary bronchial epithelial cultures upon cigarette smoke exposure and rhinovirus infection.** **(A)** Expression of type I interferon (*IFNB1*) and type III interferon (*IFNL1*) was measured by qPCR at 6, 24 and 48hpi. Data are mean values ± SEM. n=16 different donors. Analysis of differences was conducted using paired two-way ANOVA with a Tukey post-hoc test. Significant differences are indicated by P<0.05. * = AIR+RV and AIR, # = CS+RV vs CS. **(B)** Gene and pathways interaction with interferons graph at 24hpi.


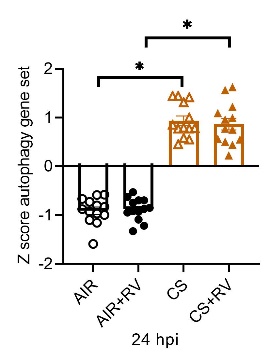

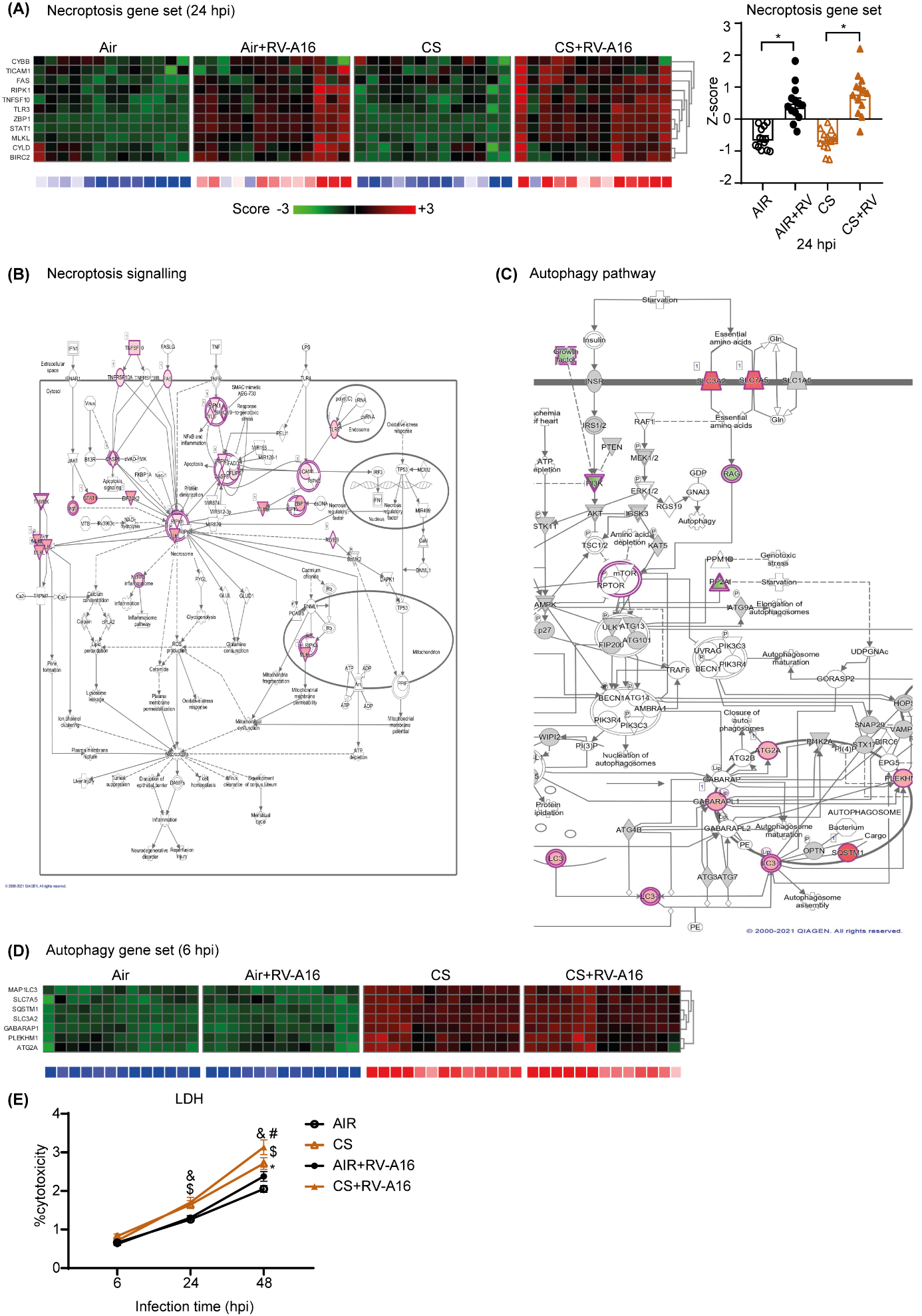


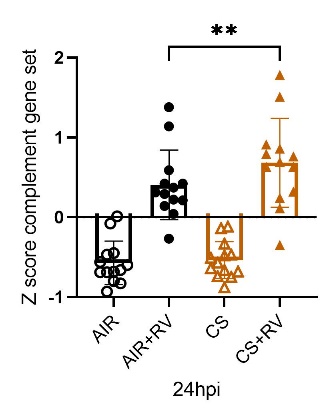

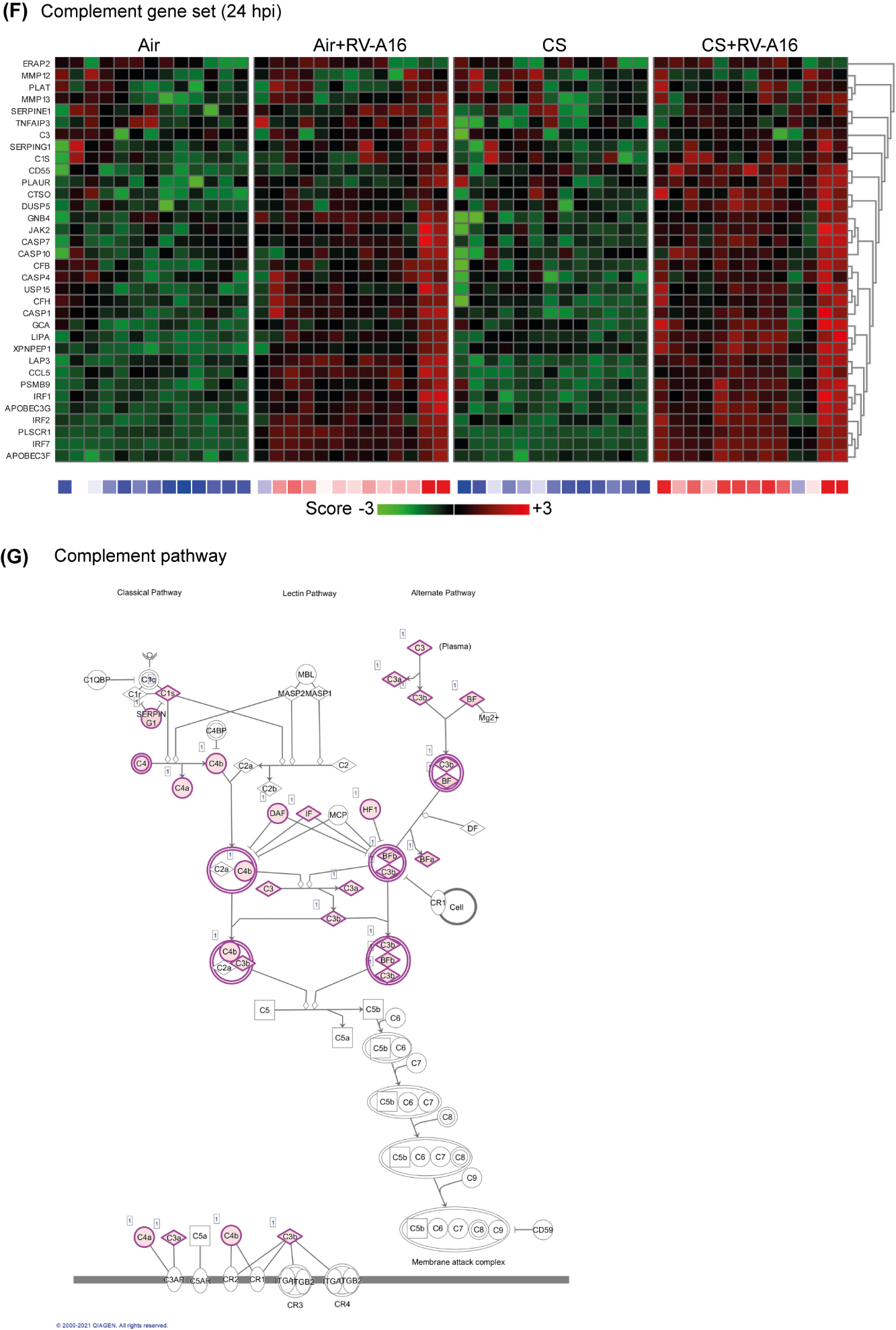


**Supplementary Figure S4. Rhinovirus-induced expression of genes related to necroptosis and complement as well as cigarette smoke-induced an increase in gene expression of autophagy.** (**A)** Heatmap showing necroptosis gene set and the average Z scores are shown. Data are shown as mean ±SEM. n*=*13 different donors. For statistical analysis, paired one-way ANOVA with a Tukey post-hoc test was used: *p<0.05. (**B and C**) The DEGs analysed using IPA showed necroptosis and autophagy pathways. **(D)** Heatmap showing autophagy gene set. **(E)** The LDH release was measured in ALI-PBEC at 6, 24 and 48hpi. Data are mean values ± SEM. n=16 different donors. Analysis of differences was conducted using paired two-way ANOVA with a Tukey post-hoc test. Significant differences are indicated by P<0.05: $ = difference between CS exposed cells and AIR controls, # = CS+RV vs CS, & = CS+RV vs AIR+RV, * = between AIR+RV and AIR. **(F)** Heatmap showing complement gene set. In all heatmaps, the Z scores for individual genes are represented in green and red, while the average Z scores (underneath the heat maps) of all genes in the gene set are represented in blue and red. The Z scores shown are relative to the average gene expression of the corresponding dataset at that time point. Below the heat maps, blue represents downregulated gene expression while red color shows upregulated gene expression. **(G)** The DEGs analysed using IPA showed necroptosis and complement pathways.


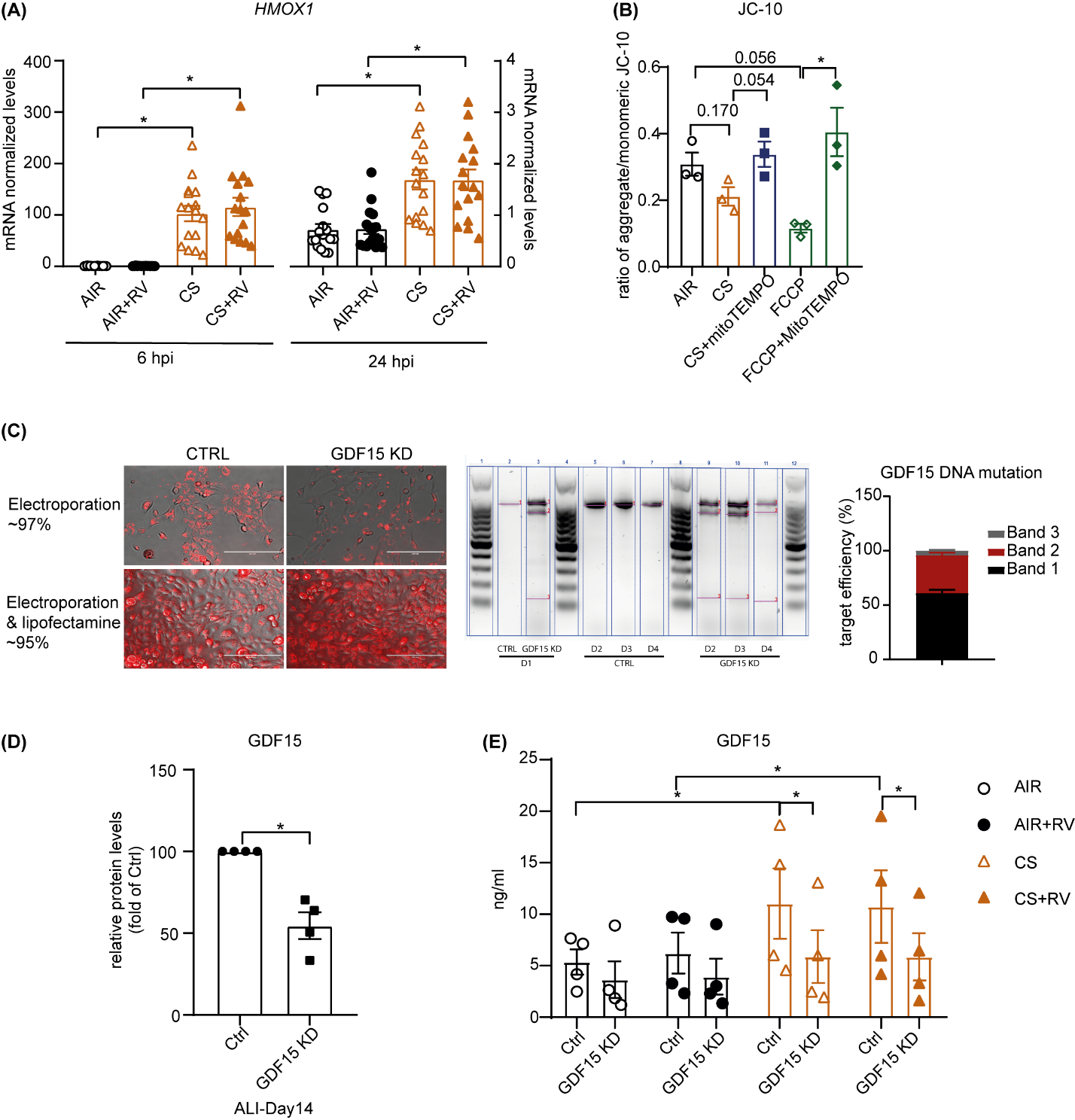


**Supplementary Figure S5. Increased oxidative stress by cigarette smoke exposure, decreased mitochondrial membrane potential by FCCP and knockdown of growth differentiation factor 15. (A)** The gene expression of *HMOX1* was measured by qPCR at 6 and 24hpi. Data are shown as mean ±SEM. n=16 different donors. For statistical analysis, paired one-way ANOVA with a Tukey post-hoc test was used: *p<0.05. **(B)** The mitochondrial membrane potential with FCCP and mitoTEMPO treatment was measured by JC-10 assay. The ratio of aggregate/monomeric JC-10 were calculated based on the fluorescence intensity. Data are shown as mean ±SEM. n=3 different donors. For statistical analysis, paired two-tailed t test was used: *p<0.05. **(C-E)** The RNP complexes including Hs.Cas9.GDF15.1.AA or Alt-R CRISPR-Cas9 Negative Control crRNA were used to transfect PBEC in submerged condition delivered by electroporation and lipofectamine transfection. Then cells were cultured on the inserts at ALI for 2 weeks. **(C)** The transfection efficiency of electroporation (after 6h of transfection) and electroporation with lipofectamine (after 24 h of lipofectamine transfection) was evaluated by counting fluorescently-labelled cells, which include tracrRNA with ATTO^TM^ 550 fluorescent dye, in control and *GDF15* knockdown cells. The target efficiency of *GDF15* knockdown cells was measured by checking for DNA mutations quantified by band intensities using Image Lab software from 4 donors by 2% agarose gel electrophoresis. **(D)** Levels of GDF15 protein were measured in basal medium of control and *GDF15* knockdown ALI-PBEC cultures after 2-weeks of differentiation, the relative protein levels of GDF15 in *GDF15* knockdown ALI-PBEC were calculated using percentages of control. For statistical analysis a paired two-tailed t-test was used: *p<0.05. **(E)** Levels of GDF15 protein were measured in basal medium after CS exposure and RV infection by ELISA at 24hpi. Data are shown as mean ±SEM. n*=*4 different donors. For statistical analysis a two-way ANOVA with Tukey’s test was used: *p<0.05.


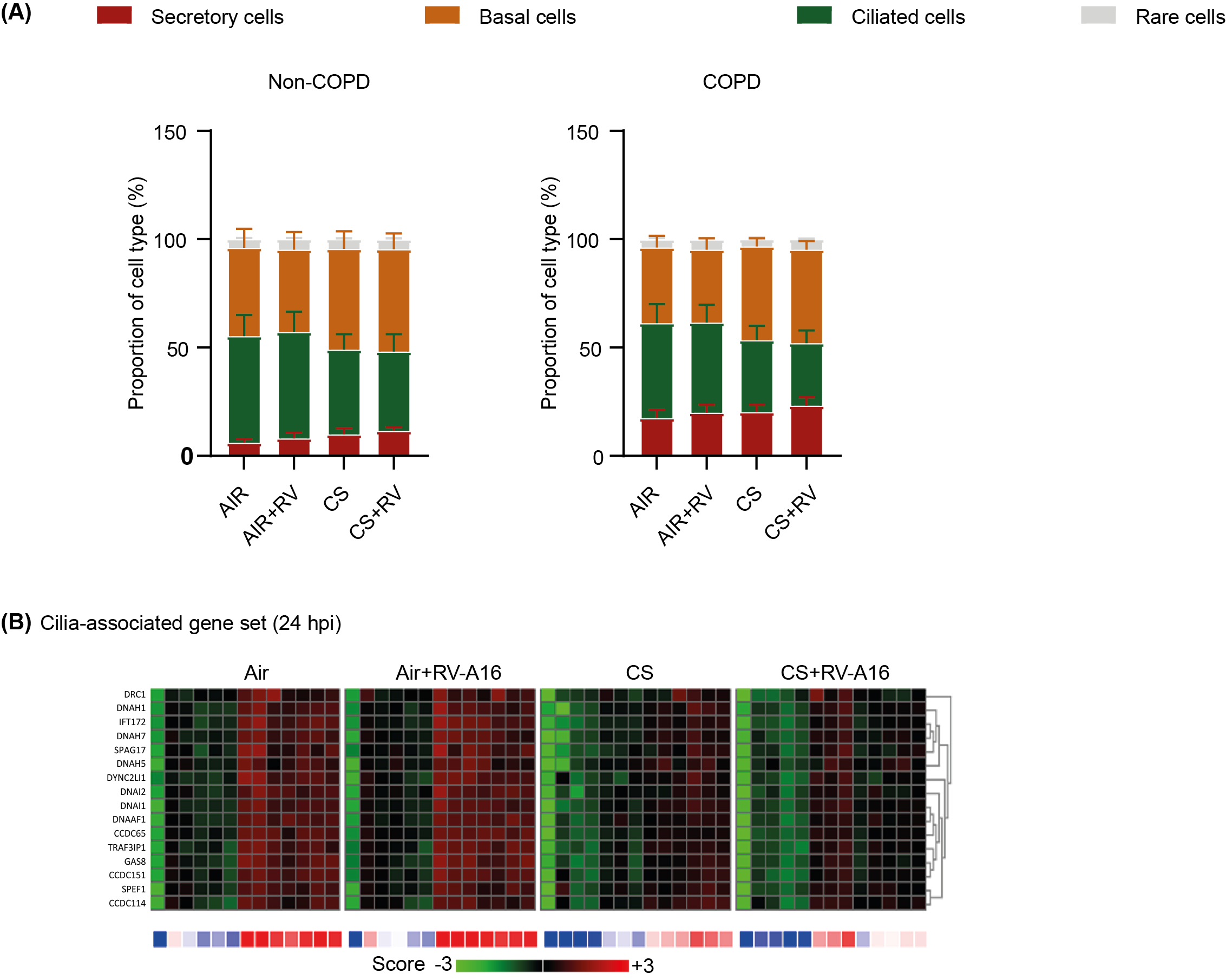


**Supplementary Figure S6.** **Relative proportion of each cell type after RV-A16 and cigarette smoke in primary human bronchial epithelial cells.** ALI-PBEC isolated from COPD and non-COPD donors were exposed to CS or air control and then directly infected with RV-A16 (MOI 1) for 1 h and next incubated for 24 h. **(A)** The relative proportion of different cell types (secretory cells, basal cells, rare cells and ciliated cells) in ALI-PBEC after CS and RV-A16 exposure as determined by cellular deconvolution of the transcriptomic datasets. **(B)** Heatmap of cilia-associated gene set is shown at 24hpi. The Z scores for individual genes are represented in green and red, while the average Z scores (below the heat maps) of all genes in the gene set are represented in blue and red. The Z scores shown are relative to the average gene expression of the corresponding dataset at that time point. Below the heat maps, the average Z scores in blue represent downregulated gene expression while red color shows upregulated gene expression.


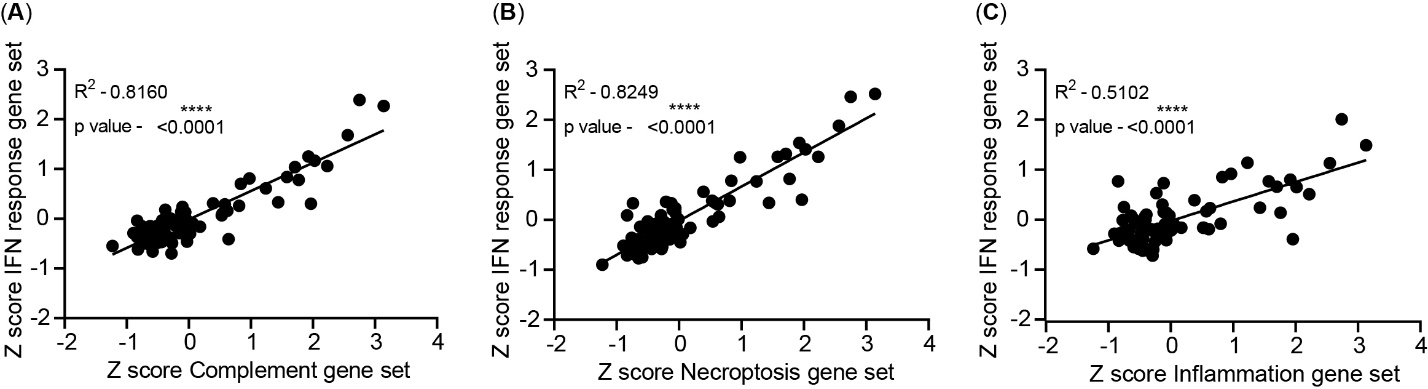


**Supplementary Figure S7. Z scores of interferon response gene set correlates with complement, necroptosis and inflammation related gene sets.** Nasal brushings were obtained from non-COPD donors (n=16) on day 3, 6, 9 and 13 after RV-A16 exposure and 1 day before as baseline. Correlations were done using Pearson correlation coefficient. Significant differences are indicated by **** P<0.0001.

**Supplementary Tables**

**Table S1. Overview of the number of differentially expressed genes (DEGs).** Gene expression following various exposures, i.e. CS exposure, RV-A16 infection and combination of CS and RV-A16, was compared to air controls. The number of differentially expressed genes was calculated based on a q value of <0.05 and a log2 fold change of >1.5.

|  | Non- COPD (6hpi) | COPD (6hpi) | Non- COPD (24hpi) | COPD (24hpi) |
| --- | --- | --- | --- | --- |
| Air vs CS | 570 | 463 | 459 | 874 |
| Air vs Air+RV | 0 | 0 | 140 | 417 |
| Air vs CS+RV | 428 | 501 | 432 | 710 |

**Table S2. Top 10 gene sets of the differentially expressed genes analyzed using gene set enrichment analysis (GSEA) and Ingenuity Pathway analysis (IPA) at 24hpi post RV-A16 infection.** The pathways highlighted in bold were selected for further analysis.

| **S.No** | **GSEA pathways (number of genes)** | **number of overlapping genes** | | | | **q-value** |
| --- | --- | --- | --- | --- | --- | --- |
| **RV-A16-exposed compared to air-exposed ALI-PBEC at 24hpi** | | | | | | |
| **1** | **Hallmark_Interferon_Gamma_Response (200)** | **92** | | | | **3.29E+132** |
| **2** | **Hallmark_Interferon_Alpha_Response (200)** | **74** | | | | **1.56E-130** |
| **3** | **Hallmark_Inflammatory_Response (200)** | **26** | | | | **1.02E-20** |
| 4 | Hallmark_TNFA_Signaling_via_NFκB (200) | 22 | | | | 4.45E-16 |
| **5** | **Hallmark_Complement (200)** | **21** | | | | **4.87E-15** |
| 6 | Hallmark_IL6_JAK_STAT3_Signaling (87) | 13 | | | | 1.70E-11 |
| 7 | Hallmark_Allograft_Rejection (200) | 17 | | | | 6.90E-11 |
| **8** | **Hallmark_Apoptosis (161)** | **15** | | | | **2.74E-10** |
| 9 | Hallmark_KRAS_Signaling_Up (200) | 16 | | | | 5.45E-10 |
| 10 | Hallmark_Hypoxia (200) | 12 | | | | 2.56E-06 |
| **S.No** | **Ingenuity pathways** | | **-log(p-value)** | **Z score** | **no. of genes** | |
| **RV-A16 exposed compared to air exposed ALI-PBEC at 24hpi** | | | | | | |
| **1** | **Interferon Signaling** | | **16.20** | **3.64** | **18** | |
| 2 | Role of Hypercytokinemia/hyperchemokinemia in the Pathogenesis of Influenza | | 15.40 | 5.00 | 25 | |
| **3** | Death Receptor Signaling | | **9.55** | **2.24** | **20** | |
| 4 | Role of Pattern Recognition Receptors in Recognition of Bacteria and Viruses | | 9.19 | 3.61 | 25 | |
| **5** | **Necroptosis Signaling Pathway** | | **7.74** | **4.38** | **23** | |
| 6 | Acute Phase Response Signaling | | 7.63 | 2.36 | 25 | |
| 7 | Systemic Lupus Erythematosus In B Cell Signaling Pathway | | 7.33 | 4.49 | 31 | |
| 8 | Neuroinflammation Signaling Pathway | | 5.62 | 4.71 | 30 | |
| **9** | **Complement System** | | **4.30** | **-0.71** | **8** | |
| **10** | **Inflammasome pathway** | | **3.18** | **2.24** | **5** | |

**Table S3. Top 10 pathway of the differentially expressed genes analyzed using gene set enrichment analysis (GSEA) and Ingenuity Pathway analysis (IPA) at 6hpi post CS exposure.** The pathways highlighted in bold were selected for further analysis.

| **S.No** | **GSEA pathways (number of genes)** | **number of overlapping genes** | | | | **q-value** |
| --- | --- | --- | --- | --- | --- | --- |
| **CS-exposed compared to air-exposed ALI-PBEC at 6hpi** | | | | | | |
| **1** | **Hallmark_Reactive_Oxygen_Species_Pathway (49)** | **8** | | | | **1.00E-06** |
| 2 | Hallmark_Xenobiotic _Metabolism (200) | 12 | | | | 1.43E-05 |
| 3 | Hallmark_UV_Response_Dn (144) | 9 | | | | 1.78E-04 |
| 4 | Hallmark_Estrogen_Response_Late (200) | 10 | | | | 1.86E-04 |
| 5 | Hallmark_Inflammatory Response (200) | **10** | | | | **1.86E-04** |
| 6 | Hallmark_KRAS_Signaling_Dn (200) | 10 | | | | 1.86E-04 |
| 7 | Hallmark_Apoptosis (161) | **9** | | | | **1.86E-04** |
| 8 | Hallmark_Estrogen_Response_Early (200) | 9 | | | | 5.34E-04 |
| **9** | **Hallmark_Interferon_Gamma_Response (200)** | **9** | | | | **5.34E-04** |
| 10 | Hallmark_KRAS_Signaling_Up (200) | 9 | | | | 5.34E-04 |
| **S.No** | **Ingenuity pathways** | | **-log(p-value)** | **Z score** | **no. of genes** | |
| **CS exposed compared to air exposed ALI-PBEC at 6hpi** | | | | | | |
| 1 | Colorectal Cancer Metastasis Signaling | | 9.73 | -1.52 | 43 | |
| 2 | Osteoarthritis Pathway | | 8.97 | 0.37 | 38 | |
| **3** | WNT/β-catenin Signaling | | 6.21 | -0.20 | 27 | |
| **4** | **Autophagy** | | **4.49** | **0.19** | **27** | |
| 5 | HMGB1 Signaling | | 4.02 | -2.50 | 22 | |
| **6** | **Role of Pattern Recognition Receptors in Recognition of Bacteria and Viruses** | | **4.00** | **-1.73** | **21** | |
| 7 | Integrin Signaling | | 2.92 | -1.89 | 23 | |
| **8** | **Oxidative Stress Response** | | **2.64** | **2,62** | **24** | |
| 9 | Xenobiotic Metabolism AHR Signaling Pathway | | 2.16 | 1,90 | 35 | |
| **10** | **Role of PKR in Interferon Induction and Antiviral Response** | | **1.49** | **-1,57** | **13** | |

**Table S4. Top 10 pathway of the differentially expressed genes analyzed using gene set enrichment analysis (GSEA) and Ingenuity Pathway analysis (IPA) at 24hpi post CS exposure.** The pathways highlighted in bold were selected for further analysis.

| **S.No** | | **GSEA pathways (number of genes)** | **number of overlapping genes** | | | | **q-value** |
| --- | --- | --- | --- | --- | --- | --- | --- |
| **CS-exposed compared to air-exposed ALI-PBEC at 24hpi** | | | | | | | |
| **1** | | **Hallmark_Reactive_Oxygen_Species_Pathway (49)** | **21** | | | | **1.54E-12** |
| 2 | | Hallmark_Xenobiotic _Metabolism (200) | 17 | | | | 6.32E-09 |
| 3 | | Hallmark_MTORC1_Signaling (200) | 13 | | | | 9.25E-06 |
| 4 | | Hallmark_Myogenesis (200) | 14 | | | | 9.25E-06 |
| 5 | | Hallmark_UV_Response_Up (158) | 11 | | | | 2.50E-05 |
| 6 | | Hallmark_Apoptosis (161) | **11** | | | | **2.50E-05** |
| 7 | | Hallmark_Complement (200) | **12** | | | | **2.50E-05** |
| 8 | | Hallmark_P53_Pathway (200) | 12 | | | | 2.50E-05 |
| 9 | | Hallmark_TNFA_Signaling_via_NFκB (200) | **12** | | | | **2.50E-05** |
| **10** | | **Hallmark_Glycolysis (200)** | **8** | | | | **6.23E-03** |
| **S.No** | **Ingenuity pathways** | | | **-log(p-value)** | **Z score** | **no. of genes** | |
| **CS exposed compared to air exposed ALI-PBEC at 24hpi** | | | | | | | |
| 1 | FAT10 Signaling Pathway | | | 6.81 | 1,00 | 24 | |
| 2 | BAG2 Signaling Pathway | | | 5.71 | -1.51 | 29 | |
| 3 | Polyamine Regulation in Colon Cancer | | | 5.25 | 0.00 | 23 | |
| 4 | p53 Signaling | | | 4.69 | 1.09 | 30 | |
| 5 | Ferroptosis Signaling Pathway | | | 3.97 | 0.87 | 34 | |
| **6** | **Oxidative Stress Response** | | | **3.43** | **2.65** | **53** | |
| 7 | Xenobiotic Metabolism Signaling | | | 3.39 | 0.00 | 62 | |
| **8** | **Glycolysis** | | | **3.37** | **2.71** | **11** | |
| 9 | Nicotine Degradation III | | | 1.55 | 0.28 | 14 | |
| **10** | **Oxidative Phosphorylation** | | | **1.53** | **4.80** | **23** | |

**Supplementary table S5. Primer sequences used for analyzing gene expression by PCR.**

| **Gene** | **Forward primer (5’-3’)** | **Reverse primer (5’-3’)** |
| --- | --- | --- |
| *ATP5B* | TCACCCAGGCTGGTTCAGA | AGTGGCCAGGGTAGGCTGAT |
| *GDF15* | TCCCTATCTGTCTTCCCACAGT | GAGGAGGATCTTCTCAAGGTCA |
| *HMOX1* | AAGACTGCGTTCCTGCTCAAC | AAAGCCCTACAGCAACTGTCG |
| *IFNB1* | ATGACCAACAAGTGTCTCCTCC | GGAATCCAAGCAAGTTGTAGCTC |
| *IFNL1* | GGACGCCTTGGAAGAGTCACT | AGAAGCCTCAGGTCCCAATTC |
| *MX1* | GTTTCCGAAGTGGACATCGCA | CTGCACAGGTTGTTCTCAGC |
| *RSAD2* | TTGGACATTCTCGCTATCTCCT | AGTGCTTTGATCTGTTCCGTC |
| *RPL13A* | AAGGTGGTGGTCGTACGCTGTG | CGGGAAGGGTTGGTGTTCATCC |
| **Virus** | **Forward primer (5’-3’)** | **Reverse primer (5’-3’)** |
| RV-A16 | ACCCTCAATACATACGCCAACT | TTCCAAGCCATCCATTCCA |

**References**

1. Amatngalim GD, Schrumpf JA, Dishchekenian F, Mertens TCJ, Ninaber DK, van der Linden AC *et al.* Aberrant epithelial differentiation by cigarette smoke dysregulates respiratory host defence. *Eur Respir J* 2018; **51**(4).

2. Wang Y, Ninaber DK, van Schadewijk A, Hiemstra PS. Tiotropium and Fluticasone Inhibit Rhinovirus-Induced Mucin Production via Multiple Mechanisms in Differentiated Airway Epithelial Cells. *Front Cell Infect Microbiol* 2020; **10:** 278.

3. Ramakrishnan MA. Determination of 50% endpoint titer using a simple formula. *World J Virol* 2016; **5**(2)**:** 85-86.
